# Loss of wbp11 causes multi-system developmental defects: a zebrafish model of VACTERL association

**DOI:** 10.3389/fcell.2026.1846751

**Published:** 2026-08-06

**Authors:** Yu Chen, Shiqi You, Yingshuo Zhang, Xueting Yang, Jinhan Li, Mei Lin, Wuzhou Yuan, Xiongwei Fan, Fang Li, Yuequn Wang, Ping Zhu, Zhigang Jiang, Yongqing Li, Xiushan Wu

**Affiliations:** 1 The Center for Heart Development, College of Life Science, Hunan Normal University, Changsha, China; 2 Medical Research Center, Guangdong Provincial People’s Hospital (Guangdong Academy of Medical Sciences), Southern Medical University, Guangzhou, China; 3 Guangdong Provincial Key Laboratory of Pathogenesis, Targeted Prevention and Treatment of Heart Disease, Guangzhou Key Laboratory of Cardiac Pathogenesis and Prevention, Guangzhou, China

**Keywords:** developmental defects, RNA splicing, VACTERL association, WBP11, zebrafish model

## Abstract

**Introduction:**

VACTERL association is a congenital disorder characterized by the non-random co-occurrence of vertebral, anal, cardiac, tracheo-esophageal, renal, and limb anomalies. *WBP11* has been identified as a candidate causative gene; however, existing heterozygous *Wbp11* knockout mice recapitulate only a subset of the patient phenotypes, most notably lacking the cardiac defects, which limits a comprehensive understanding of the pathogenic mechanisms. Whether *WBP11* loss of function is sufficient to induce the full multi-system spectrum of VACTERL association in a vertebrate model remains unexplored.

**Methods:**

We established a *wbp11* knockdown zebrafish model by microinjecting a translation-blocking morpholino (*wbp11*-MO) at the optimized concentration of 0.25 mM. Knockdown efficiency was validated by Western blot. Bioinformatics analysis of public single cell and expression atlas datasets was combined with whole-mount in situ hybridization to characterize the spatiotemporal expression of wbp11. Phenotypic defects across cardiac, vascular, spinal, fin, and renal systems were assessed by stereomicroscopy, transgenic reporter lines (Tg(*myl7:EGFP*) and Tg(*flila:EGFP*)), and whole mount in situ hybridization. Underlying molecular changes were interrogated by transcriptome sequencing (RNA-seq) coupled with differential alternative splicing analysis (rMATS) and quantitative real-time PCR validation. Rescue experiments were performed by co-injection of capped *wbp11* mRNA.

**Results:**

*wbp11* is evolutionarily conserved and maternally expressed, peaking at the gastrula stage with elevated signals in the head, skeletal muscle, and heart. *wbp11*-MO injection reduced Wbp11 protein levels, induced a 70% overall malformation rate, and caused a general delay in embryonic development. *wbp11*-deficient larvae exhibited multi-system developmental defects that closely mirror the core clinical features of VACTERL association: cardiac malformations with pericardial edema and impaired heart looping; vascular defects including underdeveloped subintestinal vessels and reduced cranial vasculature; spinal curvature with aberrant notochord structure; hypoplasia of the pectoral, pelvic, anal, and caudal fins; and transcriptional dysregulation of kidney developmental markers. Transcriptomic and alternative splicing analyses revealed significant dysregulation of genes associated with cardiac function, angiogenesis, skeletal patterning, fin development, and kidney development, accompanied by widespread splicing aberrations.

**Discussion:**

This study establishes the first *wbp11*-knockdown zebrafish model that partially recapitulates the key organ phenotypes of human VACTERL association notably including the cardiac defects absent in the mouse model and thereby provides a powerful platform for dissecting the splicing dependent molecular mechanisms underlying this disorder and for exploring potential therapeutic interventions.

## Graphical Abstract

**Figure:**
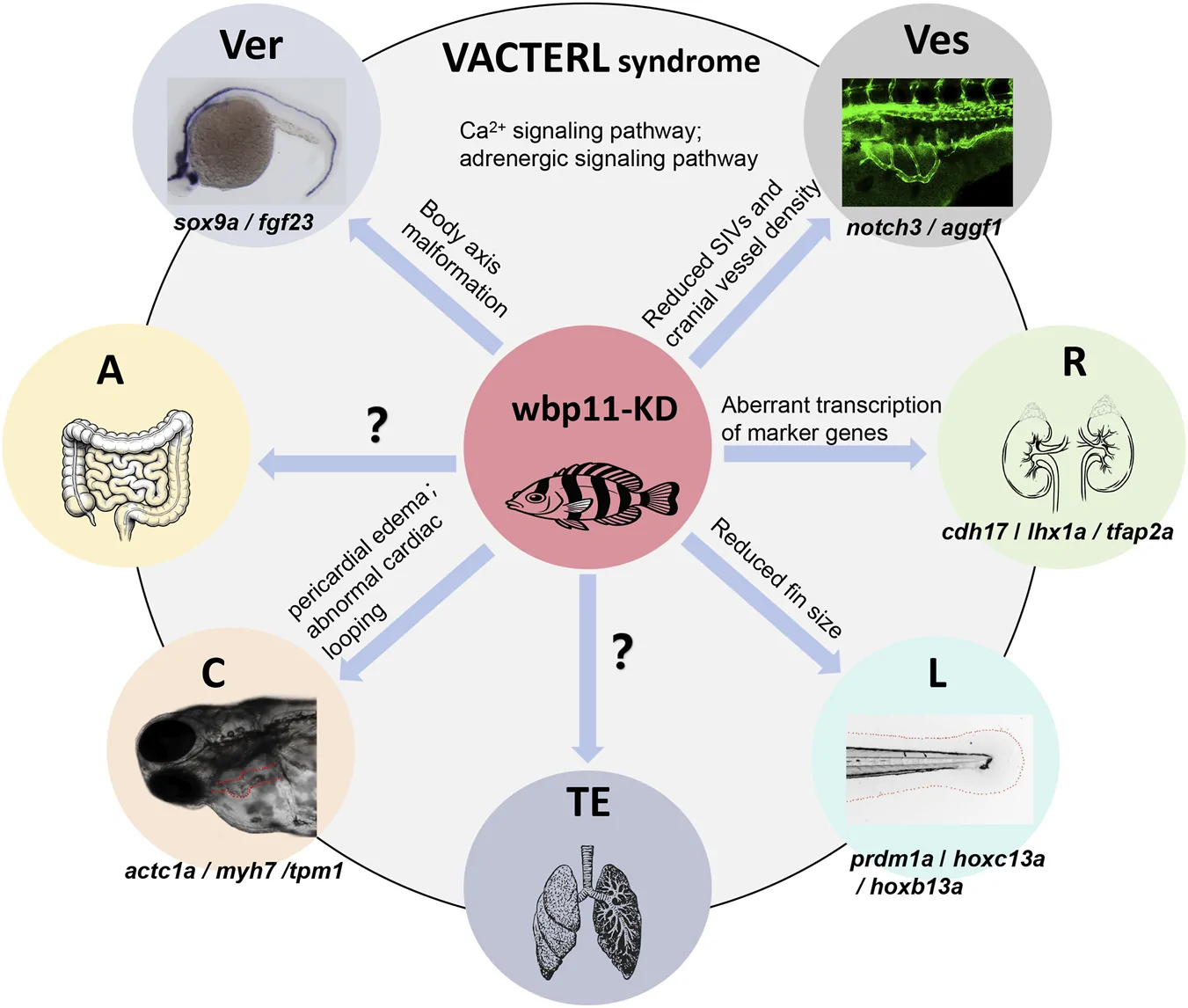
*Wbp11* deficiency causes multi-system developmental defects in zebrafish that recapitulate key features of VACTERL association. A *wbp11* knockdown zebrafish model was generated by morpholino (MO) injection, leading to significantly reduced Wbp11 protein levels. *wbp11*-deficient embryos exhibited multi-system developmental abnormalities, including cardiac defects (pericardial edema and heart looping failure), vascular anomalies (reduced mesenteric and cranial vessels), skeletal malformations (spine curvature and notochord structural defects), fin dysplasias (reduced fin area), and molecular evidence of renal impairment (transcriptional dysregulation of renal marker genes). Transcriptomic analysis revealed dysregulated expression of genes involved in heart function, angiogenesis, skeletal patterning, fin development, and renal development, providing molecular insights into the observed multi-organ defects. This zebrafish model serves as a powerful platform for dissecting the pathogenic mechanisms underlying WBP11 associated VACTERL syndrome.

## 1 Introduction

VACTERL association characterized by the non-random co-occurrence of congenital anomalies including vertebral defects, anal atresia, cardiac malformations, tracheo esophageal fistula with esophageal atresia, renal anomalies, and limb defects was first described in the early 1970s (Quan and Smith, 1973; Solomon, 2011). Following the initial reports, it was proposed that the “V” should also encompass vascular anomalies, including a single umbilical artery, while cardiac defects (“C”) were formally incorporated, leading to the description of VACTERL association as a “primary multi site developmental defect”. Subsequently, additional limb (“L”) abnormalities were suggested, and the term “VACTERL” has since become established as a descriptor for a non-random cluster of congenital malformations involving six distinct phenotypic domains, commonly referred to as VACTERL association (Temtamy and Miller, 1974). The prevalence of VACTERL association remains difficult to ascertain owing to variations in diagnostic criteria and ascertainment methods across studies (Solomon, 2011). According to data from the European Surveillance of Congenital Anomalies (EUROCAT), the birth prevalence of VACTERL association was 0.4 per 10,000 newborns between 2012 and 2017 (EUROCAT, 2019b; van de Putte et al., 2020). Previous studies have proposed three distinct, potentially interconnected, pathogenic mechanisms contributing to VACTERL association, namely, disruption of Sonic hedgehog (Shh) and Wnt signaling pathways, structural ciliary defects, and impaired ciliary signal transduction (Kim et al., 2001; Ritter et al., 2023). However, whether a single gene mutation is sufficient to recapitulate the full spectrum of key VACTERL phenotypes remains unexplored.

WW domain binding proteins constitute a family characterized by their ability to recognize and interact with WW domain-containing proteins. Members of this family, including WBP2, WBP5, and WBP11, participate in diverse cellular processes such as signal transduction, RNA splicing, and regulation of RNA polymerase activity through these interactions (Hofmann and Bucher, 1995). Among them, WW domain binding protein 11 (WBP11) contains two proline rich regions, a coiled-coil domain, and an aspartic acid rich domain, and it engages WW domain containing proteins via its proline rich motifs (Galganski et al., 2017). Studies have identified WBP11 as a novel protein essential for centriole duplication (Park et al., 2020), and it has also been implicated as an oncogenic splicing factor in ovarian cancer (Wei et al., 2024). In *Xenopus laevis*, knockdown of *wbp11* results in reduced or absent head and tail structures, mesodermal and neural developmental defects, and abnormal gastrulation, a phenotypic spectrum reminiscent of *PQBP1* depletion (Iwasaki and Thomsen, 2014). Disruption of the interaction between WBP11 and PQBP1 impairs pre-mRNA splicing in patient-derived lymphocytes, underscoring a role for WBP11 in splicing regulation (Lubs et al., 2006). Clinically, heterozygous loss-of-function variants in *WBP11* have been identified in patients with multi-system congenital anomalies closely resembling VACTERL association (Martin et al., 2020; Shin et al., 2025). Mutations in human *WBP11* give rise to multi-system congenital anomalies, including congenital heart defects, vertebral anomalies, renal malformations, and tracheoesophageal abnormalities, which closely parallel the features of VACTERL association. However, heterozygous *Wbp11* knockout mice exhibit only a subset of these defects such as vertebral anomalies, reduced brain volume, and renal abnormalities without evident cardiac malformations (Solomon et al., 2012; Martin et al., 2020). To date, the mechanisms by which *WBP11* functions during early vertebrate embryonic development and contributes to the pathogenesis of VACTERL association remain largely unexplored.

Currently, the complexity underlying the pathogenesis of VACTERL association has hindered the development of suitable disease models for in depth mechanistic studies. Zebrafish (*Danio rerio*) were first established as a model organism for developmental research in the late 1960s and have since been widely employed to investigate embryonic development and to model various human diseases (Kimmel, 1972; Adamson et al., 2018). Although *WBP11* has been proposed as a candidate causative gene for VACTERL association (Solomon et al., 2012), the molecular mechanisms by which its dysfunction contributes to the disorder remain largely unknown. To date, no zebrafish model utilizing morpholino mediated transient *wbp11* knockdown has been established to systematically investigate its role in VACTERL association related multi-organ developmental defects. Leveraging the advantages of the zebrafish system for modeling human diseases, we generated a *wbp11* knockdown model using morpholino oligonucleotides to investigate the phenotypic consequences of reduced *wbp11* expression during early embryogenesis and to explore the underlying mechanisms. This study thus provides a valuable platform for dissecting the role of *WBP11* in the pathogenesis of VACTERL association and for further mechanistic exploration of this disorder.

## 2 Methods

### 2.1 Experimental materials

Wild-type AB strain zebrafish, as well as the transgenic lines Tg (*fli1a*:EGFP) and Tg (*myl7*:EGFP), were obtained from the China Zebrafish Resource Center (CZRC) and maintained in a recirculating aquaculture system within our laboratory. The *wbp11* CRISPR/Cas9 knockout zebrafish line used for phenotypic validation was previously generated and characterized by our group (You et al., 2023). Fish were reared under constant conditions at 28.5 °C with a 14 h light/10 h dark photoperiod; the pH was maintained at approximately 7.0, and the conductivity at approximately 550 μS/cm. Adult fish were fed Artemia twice daily. Embryos used in this study were obtained through natural pairwise mating and cultured at 28 °C in embryo medium (E3 water supplemented with 0.2 μg/mL methylene blue). All animal experimental procedures were approved by the Institutional Animal Care and Use Committee.

### 2.2 Morpholino antisense oligonucleotide design and synthesis

The *wbp11* morpholino antisense oligonucleotide (*wbp11-*MO) used in this study was designed to block translation initiation by targeting the *wbp11* mRNA and it was designed and synthesized by Gene-Tools, LLC (United States). A standard Control-MO (Control-MO) was also synthesized to serve as a negative control. Lyophilized MO powder was provided at 300 nmol per tube; prior to use, the powder was briefly centrifuged and reconstituted in 300 μL of RNase-free double-distilled water to generate a 1 mM stock solution, which was stored at 4 °C. Before injection, an aliquot of the stock solution was heat-shocked at 62 °C for 2 min and allowed to cool before use. The MO sequences are listed in Table 1.

**TABLE 1:** The sequence of the MO and the *in situ* hybridization probe primers.

Primer name	Sequence (5′-3′)
Control-MO	CCT​CTT​ACC​TCA​GTT​ACA​ATT​TAT​A
*wbp11*-MO	CCG​GCA​GCG​GTC​ACC​TCT​ATT​AAT
*wbp11*-ISH-F	GTC​GCT​CTA​CTT​CGA​CTC​CG
*wbp11*-ISH-R	TAA​TAC​GAC​TCA​CTA​TAG​GGC​CAG​GAG​GCC​TCA​AGA​ATG​G
*tbx5a*-ISH-F	TGG​ATG​AGG​CAA​CTG​GTG​TC
*tbx5a*-ISH-R	CCC​TAA​TAC​GAC​TCA​CTA​TAG​GGA​CCG​GGT​ACT​CCT​TAG​TGC​T
*flk*-ISH-F	TGCCGAACATTACCTGG
*flk*-ISH-R	TAA​TAC​GAC​TCA​CTA​TAG​GGT​CGT​CCT​TCT​TCA​CCC​T
*shha*-ISH-F	CTA​CGG​CAG​AAG​AAG​ACA​T
*shha*-ISH-R	AAT​TTA​ATA​CGA​CTC​ACT​ATA​GGT​GAC​CGC​TAT​CAT​CAA​CAA
*vmhc*-ISH-F	AGC​TCC​TCC​CCA​CAT​CTT​TT
*vmhc*-ISH-R	TGT​AAT​ACG​ACT​CAC​TAT​AGG​CCT​CCC​TCT​GCT​TCT​GTT​TG
*myl7*-ISH-F	GAA​CCG​GGA​TGG​AGT​TAT​CA
*myl7*-ISH-R	TGT​AAT​ACG​ACT​CAC​TAT​AGG​GCC​CTT​AAA​CCA​AAT​GT
*wbp11*-mRNA-F	TAA​TAC​GAC​TCA​CTA​TAG​GAT​GGG​CCG​CC GCTCCACCTC
*wbp11*-mRNA-R	TCA​GAG​CAG​CCC​CTC​CAT​CTC

### 2.3 Microinjection

On the evening prior to microinjection, sexually mature zebrafish were placed into mating tanks with dividers at a male-to-female ratio of 1:1 or 2:1. The following morning, dividers were removed upon exposure to light stimulation, and fertilized eggs laid within a 15 min window were collected. The MO stock solution was diluted to the desired working concentration (0.25 mM for *wbp11*-MO, with the Control-MO adjusted to the same concentration). Microinjection needles were prepared using a micropipette puller, and approximately 1–2 nL of the MO solution was injected into the interface between the yolk sac and the cytoplasm of one-cell-stage embryos under a stereomicroscope. Following injection, embryos were transferred to Petri dishes containing E3 water and cultured in an incubator at 28 °C. The culture medium was refreshed every 12 h, and deceased embryos were removed promptly. To determine the optimal injection concentration, a preliminary experiment was performed using four concentration gradients (0.125, 0.25, 0.5, and 1.0 mM). Based on the survival rate and morphological abnormality rate at 72 h post-fertilization (hpf), 0.25 mM was selected for subsequent experiments.

### 2.4 Phenotypic observation and image acquisition of zebrafish

Embryos were collected at different developmental stages (24, 48, 72, and 96 hpf), fixed in a left lateral position using 1% low melting point agarose, and observed and imaged under a stereomicroscope (Leica, Germany). Evaluated parameters included developmental progression, pericardial edema, heart looping, spinal curvature, and fin morphology. Images were acquired using Leica imaging software and subsequently processed with Adobe Photoshop CS6.

### 2.5 Western blot

Fifty zebrafish larvae were collected from the Control-MO and *wbp11*-MO groups at 72 hpf and 96 hpf, respectively. The samples were homogenized on ice in RIPA lysis buffer (CWBio) supplemented with protease inhibitor (Roche) and phosphatase inhibitor (Roche), followed by incubation at 4 °C for 20 min. The lysates were then centrifuged at 12,000 × *g* for 15 min, and the supernatants were collected. Protein concentrations were determined using the BCA method (Thermo Scientific). The supernatants were resuspended in 5× SDS reducing loading buffer and denatured by boiling in a water bath for 10 min. Protein samples were separated by sodium dodecyl sulfate-polyacrylamide gel electrophoresis (SDS-PAGE) at a constant voltage of 120 V for 1.5 h, followed by transfer onto polyvinylidene difluoride (PVDF) membranes at a constant current of 300 mA for 60 min. The membranes were blocked with 5% non-fat milk (diluted in TBST) for 1.5 h at room temperature, then incubated with primary antibodies for 2 h at room temperature. After three washes with TBST (5 min each), the membranes were incubated with the corresponding secondary antibodies for 2 h at room temperature and washed again three times with TBST (5 min each). Protein expression was detected using a chemiluminescence imaging system (Tanon-5200Multi). The primary antibodies used were anti-WBP11 and anti-GAPDH (both from Abclonal; dilution 1:1,000). The secondary antibodies, goat anti-mouse and goat anti-rabbit, were purchased from ZEN-BIO (dilution 1:5,000). The experiment was independently repeated three times.

### 2.6 Whole-mount in situ hybridization in zebrafish embryos

Specific antisense RNA probes were designed targeting the coding sequences of zebrafish genes, including *wbp11*, *myl7*, *vmhc*, *flk*, *shha*, and *tbx5a* (primers listed in Table 1). Template fragments were amplified by PCR using a zebrafish cDNA library and subsequently purified. Digoxigenin (DIG) labeled antisense RNA probes were synthesized by *in vitro* transcription using the DIG RNA Labeling Kit (Roche). Embryos were collected at various developmental stages (0.2, 12, 24, 48, and 72 hpf) and fixed overnight at 4 °C in 4% paraformaldehyde (PFA). After washing with PBST, embryos were dehydrated through a graded methanol series and stored at −20 °C for at least 2 h. Following rehydration, embryos were digested with proteinase K (10 μg/mL) for an appropriate duration, post-fixed with 4% PFA, and pre-hybridized in pre-hybridization solution (containing 50% formamide, 5× SSC, 0.1% Tween20, 50 μg/mL heparin, and 500 μg/mL tRNA) at 65 °C for 2 h. The pre-hybridization solution was then replaced with fresh solution containing 1 μg/mL DIG labeled probe, and hybridization was carried out at 65 °C overnight. The following day, embryos were subjected to sequential washes with formamide/SSC gradients, treated with RNase A, and blocked with blocking solution. They were then incubated overnight at 4 °C with an alkaline phosphatase-conjugated anti-DIG antibody (Roche, 1:5,000). After washing, colorimetric detection was performed using NBT/BCIP staining solution (Roche) in the dark. Once staining signals were clearly visible, the reaction was terminated with PBST. Embryos were stored in glycerol and imaged using a stereomicroscope (Leica, Germany).

### 2.7 RNA extraction and quantitative real-time PCR

Zebrafish embryos injected with control-MO or *wbp11*-MO were collected at 72 hpf and homogenized in TRIzol reagent (Invitrogen). Total RNA was extracted according to the manufacturer’s instructions. RNA concentration and purity were assessed using a NanoDrop 2000 spectrophotometer (Thermo Scientific). cDNA was synthesized using a reverse transcription kit (Takara). Primers were designed to detect the mRNA expression levels of *wbp11*, *actc1a*, *myh7*, *tpm1*, *notch3*, *aggf1*, *sox9a*, *fgf23*, *prdm1a*, *hoxc13a*, *hoxb13a, cdh17, tfap2a,* and *lhx1a* (primer sequences are listed in Table 2). The quantitative real-time PCR (qPCR) program consisted of initial denaturation at 95 °C for 10 min, followed by 40 cycles of 95 °C for 10 s, 60 °C for 30 s, and 72 °C for 30 s. A melting curve analysis (60 °C–95 °C) was performed to verify the specificity of amplification. All qPCR experiments were carried out using three independent biological replicates, with each sample run in triplicate technical replicates. Zebrafish β-actin was used as an internal control for normalization. qRT-PCR was performed using 2× SYBR Green qRT-PCR Master Mix (Bimake) on a CFX96 system (Bio-Rad). Relative expression ratios of target genes between treatment and control groups were calculated using the 2^–ΔΔCt^ method. Data were analyzed and graphs were generated using GraphPad Prism 6 software.

**TABLE 2:** Primers for qPCR.

Primer name	Sequence (5′-3′)
z-*gapdh*-F	CCA​CCC​ATG​GAA​AGT​ACA​AG
z-*gapdh*-R	CTC​TCT​TTG​CAC​CAC​CCT​TA
z-*wbp11*-236-F	CAT​GGA​GAA​GCT​GGA​CGA​GAT​GG
z-*wbp11*-236-R	CCA​CGG​ACT​CTG​CAT​TCT​TGA​CG
z-*sox9a*-164-F	TCT​GGG​AAA​ACT​TTG​GAG​ATT​ACT​G
z-*sox9a*-164-R	TGC​CGT​CTT​CAG​ATT​CGC​TC
z-*fgf23*-248-F	CGG​GGC​TCA​TAC​AGT​GTA​ATC
z-*fgf23*-248-R	TGT​CCC​GCT​ATA​AAC​GCC​TG
z-*notch3*-119-F	ACT​GCT​CAC​ACC​TTG​CTC​TC
z-*notch3*-119-R	TGT​TGC​ACA​AAT​GGC​CTT​GC
z-*tpm1*-213-F	CAC​TCA​GCG​TCT​CCG​GTT​TG
z-*tpm1-*213-R	CAC​ATC​ACC​TTC​AGC​TGT​TTC​T
z-*myh7*-263-F	CTT​GGT​GCA​CAT​CAG​ACA​AGG
z-*myh7*-263-R	CTG​GGG​GTG​AAT​GTC​AGC​TT
z-*prdm1a*-194-F	GAT​GCA​AAA​ACT​CAA​GCA​GAG​C
z-*prdm1a*-194-R	GTG​GAT​AGA​CAG​CAC​TGG​GG
z-*actc1a*-193-F	ACT​CCA​GTC​TTG​CGC​TAC​AA
z-*actc1a*-193-R	TAC​CAA​CCA​TCA​CAC​CCT​GG
z-*aggf1*-186-F	GAA​AGA​GCT​TCC​TGT​CCC​GA
z-*aggf1*-186-R	TCG​TTT​TCT​GCT​TCG​GTC​AC
z-*hoxc13a*-141-F	CGT​CAC​GAT​GCA​TTG​ATC​CC
z-*hoxc13a*-141-R	CAA​CGG​AAC​AAC​ATC​TGG​AAA​G
z-*hoxb13a*-127-F	CCT​GCC​AAT​GGA​CAG​CTA​CC
z-*hoxb13a*-127-R	GTG​GGC​CAC​AAC​ATC​TGC​TAA​T
z-*cdh17*-160-F	CTC​GTC​AGC​ATT​GGT​CAT​GG
z-*cdh17*-160-R	CGG​TCT​CTC​CGC​TTA​CAT​GG
z-*tfap2a*-179-F	AAA​ATG​GGA​GAC​TGG​CAG​GAT​CG
z-*tfap2a*-179-R	GAC​TGC​GGA​TAG​ATG​GGC​TG
z-*lhx1a*-207-F	TGT​GGG​AGC​ACA​TCC​AAA​GA
z-*lhx1a*-207-R	TTT​GTT​CCA​AAG​CGC​CTA​AAG​A

### 2.8 Transcriptome analysis

Zebrafish embryos at 72 hpf from the Control-MO and *wbp11*-MO groups were collected, with three biological replicates per group and 50 embryos per replicate. Samples were preserved in RNAlater (Servicebio) and stored at −80 °C prior to being shipped on dry ice to Shanghai Personal Biotechnology Co., Ltd. for library construction, transcriptome sequencing, and bioinformatics analysis. mRNA with polyA tails was enriched from total RNA using Oligo (dT) magnetic beads and fragmented to approximately 300 bp using an ion-based fragmentation method. Following library construction, PCR amplification was performed to enrich the library fragments, after which size selection was conducted to obtain libraries of approximately 450 bp. Library quality was assessed using an Agilent 2100 Bioanalyzer, and both total concentration and effective concentration of the libraries were determined. Transcriptome libraries were sequenced on a HiSeq 2500 sequencing system (Illumina). Raw sequencing reads were subjected to quality filtering, and the resulting clean reads were aligned to the zebrafish reference genome. Gene expression levels were quantified using HTSeq-count and normalized as fragments per kilobase of transcript per million mapped reads (FPKM). Differentially expressed genes were identified using the DESeq2 package with thresholds of |log2 (fold change)| ≥ 1 and adjusted p < 0.05. Gene Ontology (GO) functional annotation and Kyoto Encyclopedia of Genes and Genomes (KEGG) pathway enrichment analyses were performed using the clusterProfiler R package, with adjusted *: p < 0.05 considered statistically significant. To identify splicing events affected by *wbp11* knockdown, differential alternative splicing analysis was performed using rMATS (v4.1.0) with default parameters (Shen et al., 2014). Five types of alternative splicing events were analyzed: skipped exons (SE), alternative 5′ splice sites (A5SS), alternative 3′ splice sites (A3SS), mutually exclusive exons (MXE), and retained introns (RI). Events with |ΔIncLevel| ≥ 0.1 and FDR < 0.05 were considered significantly differentially spliced.

### 2.9 *In Vitro* synthesis of capped *wbp11* mRNA and rescue microinjection

The coding sequence (CDS) of zebrafish *wbp11* was amplified by PCR using gene-specific primers containing the T7 promoter sequence at their 5′ ends (Table 1). The purified PCR product was cloned into the pESI-T simple vector (Yeasen) via TA ligation. After sequence verification, the recombinant plasmid was propagated in *E. coli*, linearized by restriction digestion with *Not*I, and used as a large-scale template for *in vitro* transcription. Capped mRNA was synthesized *in vitro* using the T7 mMessage mMachine kit (Ambion) following the manufacturer’s protocol. The resulting mRNA was purified with the RNeasy Mini Kit (Qiagen), quantified, and diluted to the working concentration in RNase-free water containing 0.05% phenol red. For rescue assays, *wbp11*-MO was co-injected with the capped *wbp11* mRNA into one-cell stage embryos. Microinjection was performed using pulled glass micropipettes with a calibrated injection volume of approximately 1 nL per embryo. Co-injected embryos were incubated at 28.5 °C in E3 medium and scored for phenotypic rescue at appropriate developmental stages.

### 2.10 Statistical analysis

All qRT-PCR data were obtained from three independent experiments, each performed in triplicate. Graphical representations were generated using Adobe Photoshop CS6 and GraphPad Prism 6 software. Western blot band intensities were quantified using ImageJ software. Data are presented as the mean ± standard error of the mean (SEM) from three independent experiments. For comparisons between two groups, two-tailed Student’s t-test (or Welch’s t-test when variances were unequal) was used for normally distributed data; otherwise, Mann-Whitney U test was applied. For comparisons involving more than two groups, One-Way ANOVA followed by Tukey’s HSD or Dunnett’s *post hoc* test was used. For the fin area analysis, Two-Way ANOVA with treatment group and fin type as factors was performed, followed by *post hoc* comparisons with Bonferroni correction for multiple testing. Significance levels are indicated as *: p < 0.05, **: p < 0.01, and ***: p < 0.001.

## 3 Results

### 3.1 Expression pattern of *wbp11* during early zebrafish development and its evolutionary conservation

**FIGURE 1:**
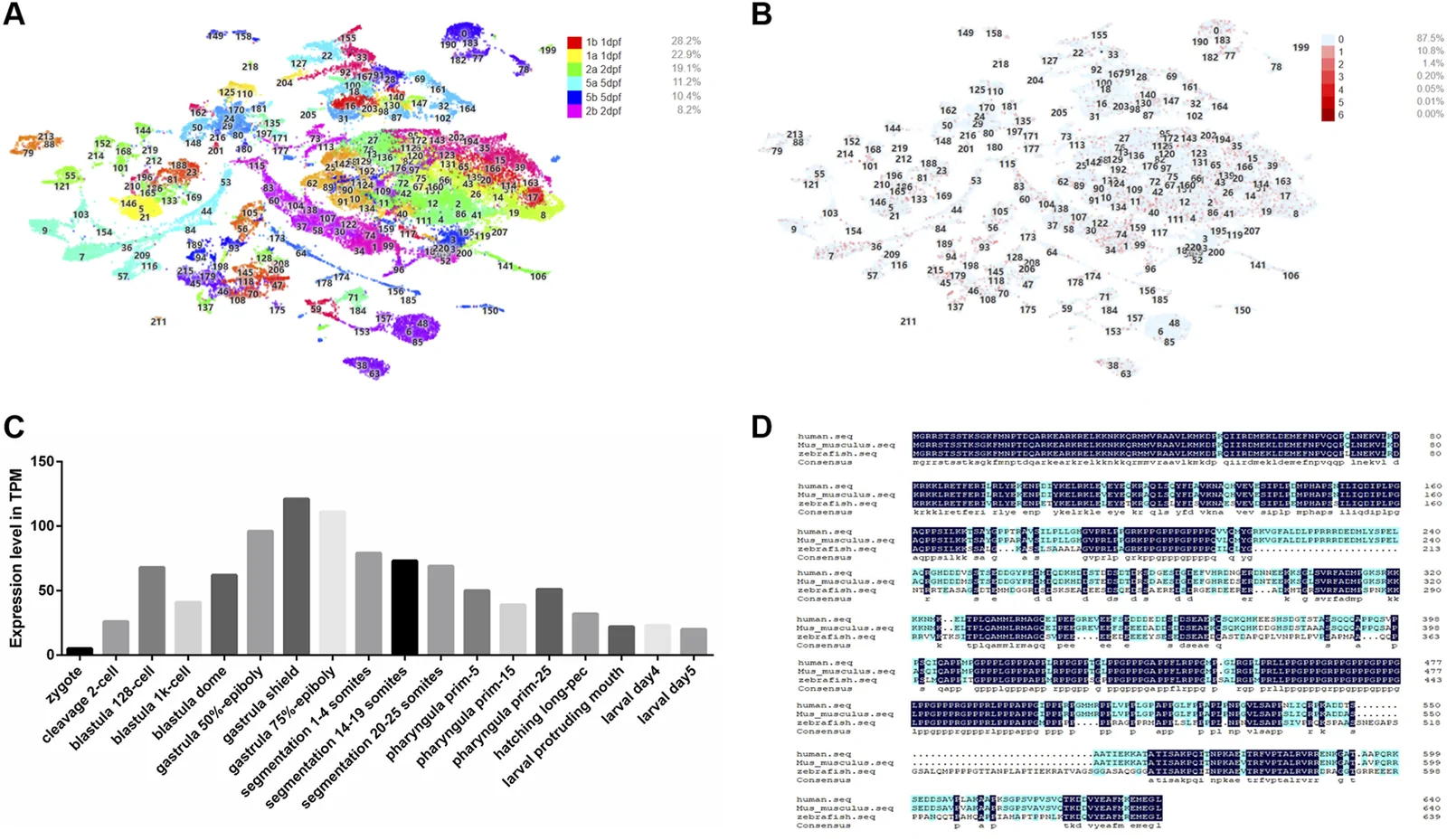
Bioinformatics analysis of *wbp11*. **(A)** UMAP clustering plots at different developmental stages (1 dpf, 2 dpf, and 5 dpf) retrieved from the UCSC Cell Browser platform. Different colors represent distinct clusters. **(B)** UMAP plot showing *wbp11* gene expression. **(C)** Expression levels of zebrafish *wbp11* at various stages of early embryonic development retrieved from the Expression Atlas database. TPM (transcripts per million) represents normalized gene expression counts calculated from raw sequencing data. **(D)** Amino acid sequence alignment of WBP11 across different species, including human, mouse, and zebrafish.

To investigate the potential role of *wbp11* during early zebrafish development, we first analyzed its expression pattern across various tissues and developmental stages. Single cell transcriptomic data from zebrafish embryos at 1, 2, and 5 dpf, accessed through the UCSC Cell Browser platform, revealed that *wbp11* was expressed across all examined tissues and organs at the three stages (Figures 1A,B), indicating that it is a ubiquitously expressed gene. Expression data for zebrafish *wbp11* at different developmental stages, retrieved from the Expression Atlas database, showed that *wbp11* transcripts were already present in fertilized eggs, peaked at the gastrula stage, and continued to be expressed through 5 dpf (Figure 1C). Amino acid sequence alignment of WBP11 from human, mouse, and zebrafish using DNAMAN software demonstrated a high degree of similarity among the three species (Figure 1D), suggesting that wbp11 is evolutionarily conserved in its function.

**FIGURE 2:**
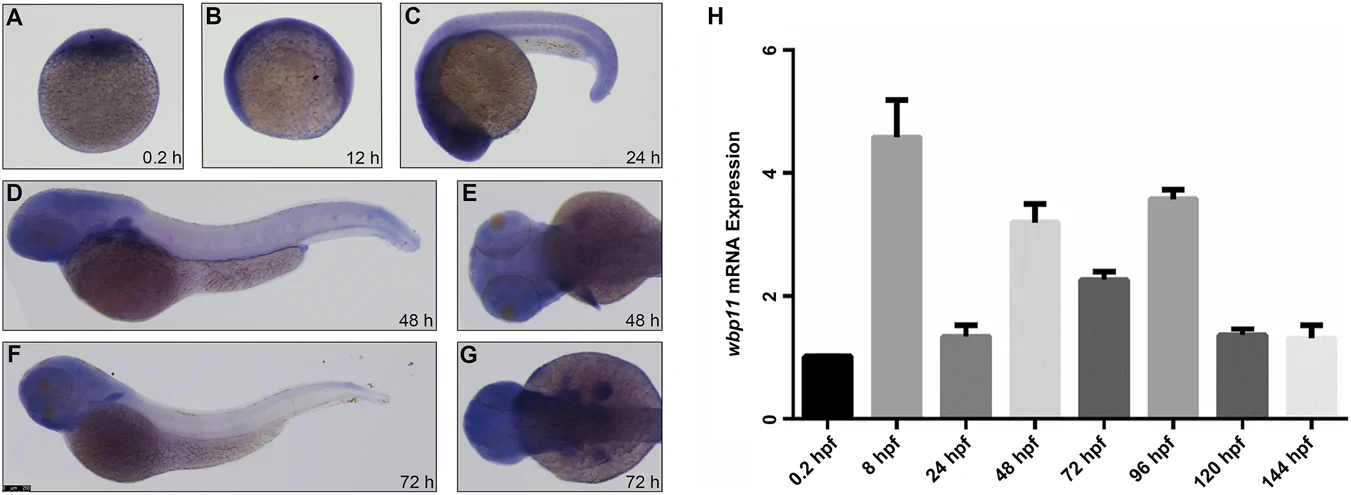
Expression profiling of zebrafish *wbp11* at different developmental stages. **(A–G)** Spatiotemporal expression of *wbp11* detected by whole-mount *in situ* hybridization in zebrafish embryos. **(A)** 0.2 hpf; **(B)** 12 hpf; **(C)** 24 hpf; **(D)** 48 hpf; **(E)** 48 hpf (dorsal view); **(F)** 72 hpf; **(G)** 72 hpf (dorsal view). **(H)** Quantitative real time PCR (qRT-PCR) analysis of *wbp11* expression levels in zebrafish embryos at indicated developmental stages. Scale bar: 250 μm.

To further characterize the spatiotemporal expression pattern of *wbp11* during zebrafish embryonic development, whole-mount *in situ* hybridization was performed on embryos collected at five developmental stages: 0.2 hpf, 12 hpf, 24 hpf, 48 hpf, and 72 hpf. The results showed that *wbp11* was broadly expressed at the zygote stage (0.2 hpf) and during gastrulation (12 hpf) (Figures 2A,B). At 24 hpf, *wbp11* expression remained widespread; however, hybridization signals were markedly enhanced in the head and skeletal muscle, whereas no expression was detected in the heart, which had not yet formed at this stage (Figure 2C). By 48 hpf, *wbp11* expression was predominantly localized to the head, with weak expression observed in the heart and reduced signal in the tail compared to 24 hpf (Figures 2D,E). At 72 hpf, distinct hybridization signals were detected in the head and the pectoral fin buds (Figures 2F,G). Thus,*wbp11* is broadly expressed across multiple tissues during early embryogenesis, with relatively higher transcript abundance detected in the head, skeletal muscle, and pectoral fin bud regions by WISH (Figures 2A–G). These findings suggest that *wbp11* may be involved in the regulation of multiple organ systems, including the head, skeletal muscle, heart, and fins. Quantitative real-time PCR was subsequently performed to examine *wbp11* expression levels at eight time points: 0.2, 8, 24, 48, 72, 96, 120, and 144 hpf. The results demonstrated that *wbp11* was expressed as early as 0.2 hpf and persisted through 144 hpf, with expression peaking at 8 hpf (gastrula stage) (Figure 2H), consistent with the findings from the database analysis.

### 3.2 Knockdown of *wbp11* leads to developmental delay and increased malformation rate in zebrafish embryos

**FIGURE 3:**
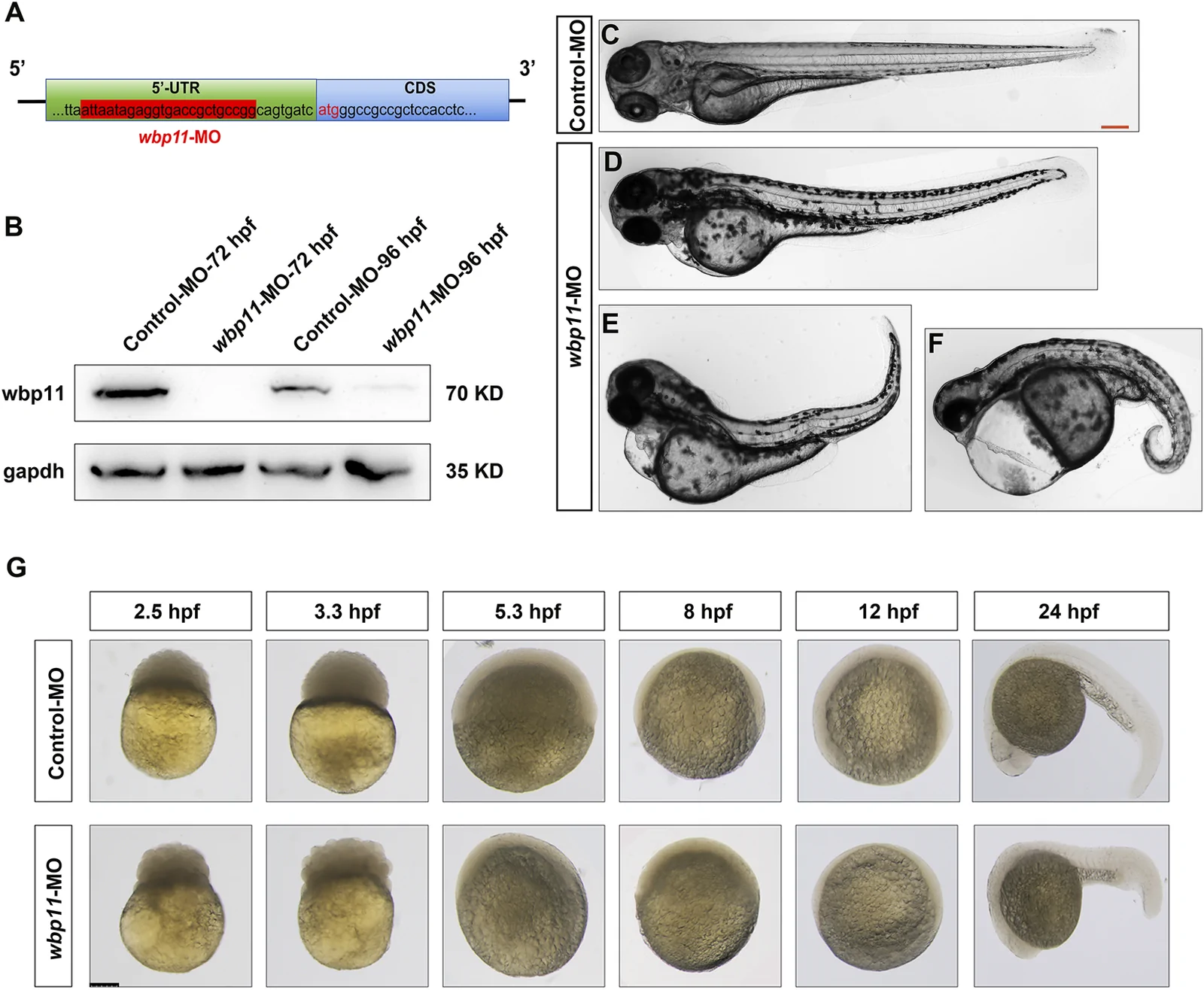
Validation of *wbp11* knockdown efficiency using morpholino in zebrafish. **(A)** Schematic diagram illustrating the targeting site of *wbp11*-MO. **(B)** Western blot analysis of wbp11 protein expression in zebrafish embryos at 72 hpf and 96 hpf to assess *wbp11*-MO knockdown efficiency. **(C)** Representative bright-field image of a zebrafish larva at 72 hpf following injection with Control-MO. **(D–F)** Representative bright-field images of zebrafish larvae at 72 hpf following injection with *wbp11*-MO, showing mild **(D)**, moderate **(E)**, and severe **(F)** malformation phenotypes. **(G)** Comparative bright-field observation of early embryonic development (2.5–24 hpf) in zebrafish embryos injected at the one-cell stage with Control-MO or *wbp11*-MO. Black scale bar: 250 μm; red scale bar: 100 μm (applies to **C–F**).

To investigate the biological function of wbp11, we designed and synthesized a morpholino antisense oligonucleotide targeting the translation start site of *wbp11* mRNA (*wbp11*-MO) to inhibit its protein translation (Figure 3A). A preliminary concentration gradient experiment established 0.25 mM as the optimal injection concentration, at which embryos exhibited a relatively high survival rate together with a significant malformation rate (Table 3). To assess knockdown efficiency, larvae were collected at 72 hpf and 96 hpf following injection of either Control-MO or *wbp11*-MO and subjected to Western blot analysis. The results revealed that wbp11 protein levels were markedly reduced in the *wbp11*-MO group compared with the Control-MO group (*: p < 0.05) (Figure 3B; Supplementary Figures S1A,B), indicating that *wbp11*-MO effectively suppressed translation of the target gene. Phenotypic observation of zebrafish embryos at 72 hpf after injection with Control-MO or *wbp11*-MO showed that the overall malformation rate in the *wbp11*-MO group was approximately 70%, significantly higher than that in the Control-MO group (Table 4; Supplementary Figure S1C). Based on the severity of the malformations, embryos in the *wbp11*-MO group were categorized into mild, moderate, and severe classes; among these, the moderate category accounted for the highest proportion, followed by the severe category, while the mild category represented the smallest proportion (Figures 3C–F; Table 5; Supplementary Figure S1D). Additionally, analysis of developmental progression revealed that, starting at 2.5 hpf, embryos in the *wbp11*-MO group exhibited a significantly slower developmental rate compared with those in the Control-MO group, and this developmental delay persisted through subsequent stages (Figure 3G). This phenotype was confirmed to be specific to *wbp11* deficiency, as co-injection of *wbp11* mRNA significantly decreased the proportion of severely affected embryos and increased the proportion of phenotypically normal embryos, thereby demonstrating effective phenotypic rescue (Table 6; Supplementary Figure S2; Supplementary File S1). Further corroborating these findings, both homozygous and heterozygous *wbp11* CRISPR/Cas9 mutants exhibited pericardial edema, cardiac linearization, and spinal curvature, consistent with the MO-induced phenotypes (You et al., 2023; Supplementary File S2). Collectively, these findings indicate that *wbp11* plays an essential role during early embryonic development in zebrafish.

**TABLE 3:** Verifcation of *wbp11*-MO concentration gradient.

Group	Injecfon concentration	Number of embryos injected	Embryo surviva rate (%)	Embryo malformation rate (%)
*wbp11*-MO	1 mmol/L	170	12.35	89.42
	0.75 mmol/L	138	25.36	82.61
	0.5 mmol/L	247	43.04	78.54
	0.3 mmol/L	194	59.43	75.62
	0.25 mmol/L	236	69.41	71.19
	0.1 mmol/L	213	70.06	53.17
Control-MO	0.25 mmol/L	178	9.78	13.48

**TABLE 4:** Statistics on overall embryo malformation rate after MO injection.

Group	Total malformation rate (%)
Control-MO	13.48
	12.65
	10.97
*wbp11*-MO	71.19
	68.80
	70.48

Data were pooled from three independent injection experiments. Total N = 590 embryos for the Control-MO group and N = 632 embryos for the *wbp11*-MO group.

**TABLE 5:** Statistics on the degree of deformity after injection of *wbp11*-MO.

Group	Mild deformity (%)	Moderate deformity (%)	Severe deformity (%)
*wbp11*-MO	18.45	57.74	23.81
	24.41	44.09	31.50
	18.25	54.74	27.01

Data were pooled from three independent injection experiments. Total N = 632 embryos for the *wbp11*-MO group.

**TABLE 6:** Classification of morphological defects at 72 hpf after *wbp11* knockdown and rescue.

Group	Total deformed (n)	Mild	Moderate	Severe	No obvious phenotype
Control-MO	11	7	2	2	132
*wbp11*-MO	85	28	37	20	17
*wbp11*-MO + mRNA (rescue)	25	12	8	5	79

Severity distribution of morphological abnormalities in wbp11 morphants at 72 hpf. Embryos were categorized into four groups based on gross morphology under stereomicroscope. Severity criteria (used for classification): Mild: slight spinal curvature, minor reduction of fin area, slightly enlarged pericardium. Moderate: obvious spinal curvature, reduced fin area, distinct pericardial edema. Severe: severe spinal curvature, pronounced fin atrophy, severe cardiac malformation.

### 3.3 Transcriptome sequencing reveals disruption of multiple signaling pathways upon *wbp11* knockdown

**FIGURE 4:**
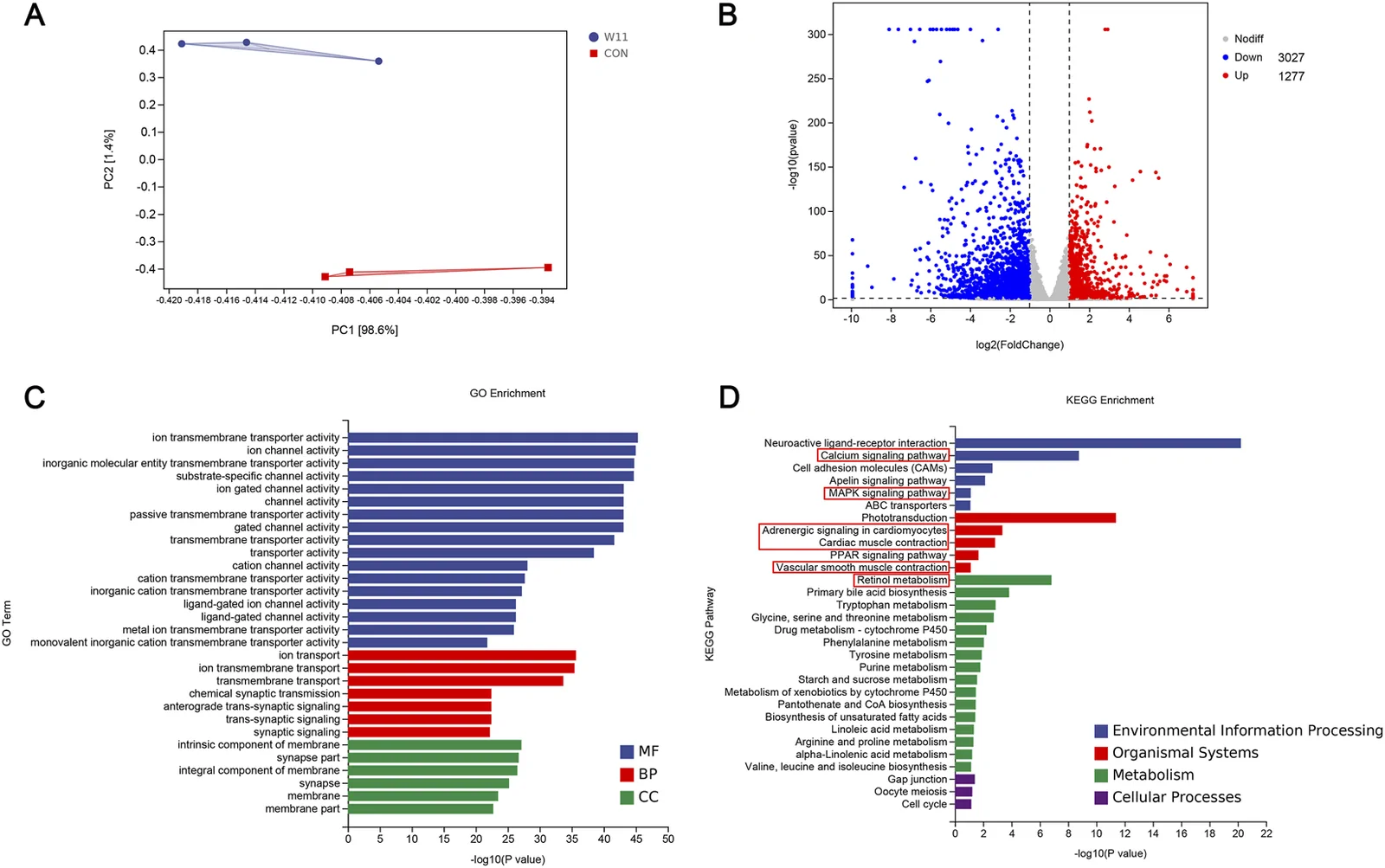
Transcriptome analysis of zebrafish embryos at 72 hpf following *wbp11*-MO knockdown. **(A)** Principal component analysis (PCA) of RNA-seq data. The horizontal axis represents the first principal component (PC1), and the vertical axis represents the second principal component (PC2). Each dot corresponds to an individual sample, with colors indicating different groups (red: Control-MO; blue: *wbp11*-MO). **(B)** Volcano plot of differentially expressed genes (DEGs). Blue dots represent significantly downregulated genes (n = 3,027), red dots represent significantly upregulated genes (n = 1,277), and gray dots represent genes with no significant differential expression. **(C)** Gene Ontology (GO) enrichment analysis of DEGs (top 30 terms). MF: molecular function; BP: biological process; CC: cellular component. **(D)** Kyoto Encyclopedia of Genes and Genomes (KEGG) pathway enrichment analysis of DEGs (top 30 pathways).

To investigate the molecular mechanisms underlying the developmental defects caused by *wbp11* knockdown, we performed transcriptome sequencing on larvae collected at 72 hpf after injection with Control-MO or *wbp11*-MO. Principal component analysis (PCA) showed high reproducibility within groups and clear segregation between groups (Figure 4A). Volcano plot analysis identified a total of 4,304 differentially expressed genes (DEGs), among which 1,277 were upregulated and 3,027 were downregulated (Figure 4B). Gene Ontology (GO) enrichment analysis revealed that the DEGs were primarily involved in molecular functions such as ion channel activity, membrane composition, and extracellular signal transduction (Figure 4C). To identify significantly enriched signaling pathways in the context of the whole genome, Kyoto Encyclopedia of Genes and Genomes (KEGG) pathway enrichment analysis was performed. The results showed that *wbp11* knockdown significantly affected pathways including neuroactive ligand receptor interaction, calcium signaling pathway, adrenergic signaling in cardiomyocytes, and retinol metabolism (Figure 4D). To determine whether these transcriptomic changes are a direct consequence of splicing failure, we performed differential alternative splicing analysis on the same RNA-seq data. This analysis identified widespread alternative splicing events, including intron retention and exon skipping, upon *wbp11* knockdown (Supplementary Figure S3), providing direct evidence that the splicing function of wbp11 is indeed affected in the morphants. Collectively, these results suggest that *wbp11* may regulate the expression of genes involved in multiple signaling pathways, thereby influencing the proper development of multiple organs, including the heart, vasculature, spine, and fins.

### 3.4 Knockdown of *wbp11* leads to cardiac developmental defects in zebrafish

**FIGURE 5:**
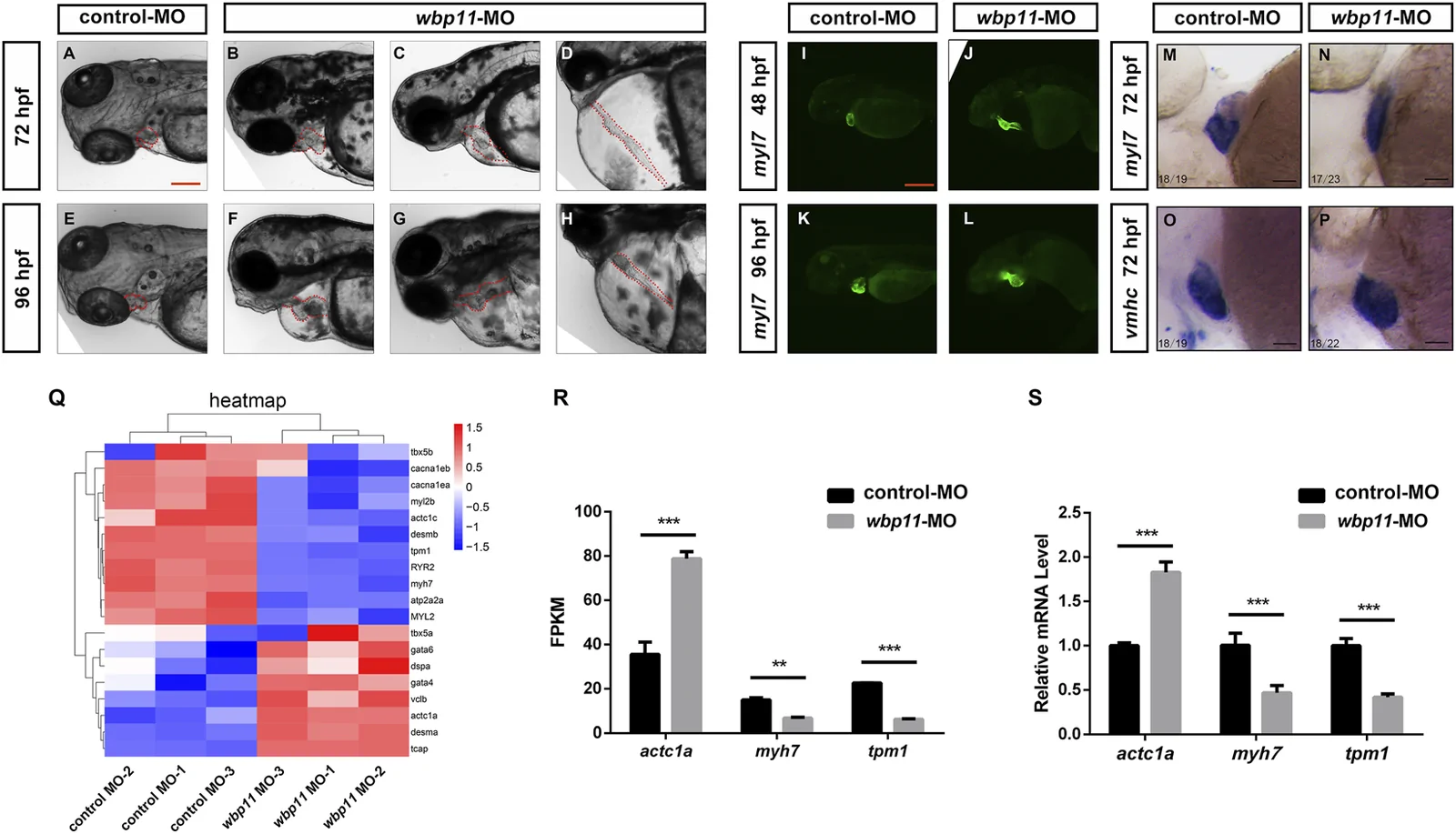
Knockdown of *wbp11* leads to cardiac defects in zebrafish. **(A–H)** Bright-field microscopy observation of cardiac morphology in zebrafish larvae. **(A–D)** 72 hpf; **(E–H)** 96 hpf. **(A,E)** Control-MO; **(B–D,F–H)** *wbp11*-MO. **(I–L)** Fluorescence microscopy observation of cardiac morphology in *Tg(myl7:EGFP)* zebrafish larvae. **(I,J)** 48 hpf; **(K,L)** 96 hpf. Red scale bar: 200 μm (applies to **(A–L)**). **(M–P)** Whole-mount *in situ* hybridization of zebrafish larvae at 72 hpf using *myl7* and *vmhc* probes to assess cardiac morphology. Black scale bar: 125 μm (applies to **(M–P)**). **(Q)** Heatmap analysis of cardiac-related differentially expressed genes (DEGs). Blue indicates downregulated genes, and red indicates upregulated genes. **(R)** FPKM values of representative cardiac genes *actc1a*, *myh7*, and *tpm1* (n = 3; biologically independent experiments, each performed with 50 pooled embryos per group. Data are presented as mean ± SEM. **p < 0.01, ***p < 0.001). **(S)** qPCR analysis of transcript levels of representative cardiac genes *actc1a*, *myh7*, and *tpm1* (n = 3; biologically independent experiments, each performed with 50 pooled embryos per group. Data are presented as mean ± SEM. ***p < 0.001).

To assess the impact of *wbp11* knockdown on cardiac development, we first performed morphological observations of larvae at 72 hpf and 96 hpf under a stereomicroscope. The results revealed that the majority of embryos in the *wbp11*-MO group exhibited malformations including pericardial edema, abnormal heart looping, and cardiac linearization (Figures 5A–H; Table 7; Supplementary Figure S4A). To visualize cardiac morphology more directly, we utilized the Tg (*myl7*:EGFP) transgenic line, which labels cardiomyocytes, and confirmed that embryos injected with *wbp11*-MO showed impaired heart looping and abnormal alignment of the atria and ventricles at 48 hpf and 96 hpf (Figures 5I–L). Whole-mount *in situ* hybridization for the cardiac marker genes *myl7* (labeling both atria and ventricles) and *vmhc* (labeling the ventricle) demonstrated that, compared to the Control-MO group, embryos in the *wbp11*-MO group exhibited diminished myl7 signals in the cardiac region with blurred boundaries between atria and ventricles (Figures 5M,N); conversely, the ventricular region labeled by *vmhc* appeared relatively enlarged (Figures 5O,P), suggesting that ventricular specification may be affected. Heatmap analysis of differentially expressed genes (DEGs) related to cardiac development derived from the transcriptome data revealed significant expression changes in multiple key cardiac developmental genes following *wbp11* knockdown (Figure 5Q). Expression levels of the cardiac contraction-related genes *actc1a*, *myh7*, and *tpm1* were further analyzed using FPKM values and validated by qPCR. The results showed that actc1a expression was significantly upregulated in the *wbp11*-MO group compared to the Control-MO group, whereas *myh7* and *tpm1* were significantly downregulated (Figures 5R,S), consistent with the transcriptome sequencing data. Furthermore, co-injection of *wbp11* mRNA reversed the transcriptional changes of *actc1a*, *myh7*, and *tpm1* in the *wbp11*-MO group (Supplementary Figure S5). Collectively, these findings demonstrate that *wbp11* knockdown disrupts both cardiac morphogenesis and the underlying molecular regulatory networks.

**TABLE 7:** Statistics on cardiac malformations rate after MO injection.

Group	Proportion of cardiac malformations in abnormal embryos (%)
Control-MO	8.33
	4.76
	7.69
*wbp11*-MO	84.52
	83.46
	84.67

Data were pooled from three independent injection experiments. Total N = 590 embryos for the Control-MO group and N = 632 embryos for the *wbp11*-MO group.

### 3.5 Knockdown of *wbp11* causes vascular developmental defects in zebrafish

**FIGURE 6:**
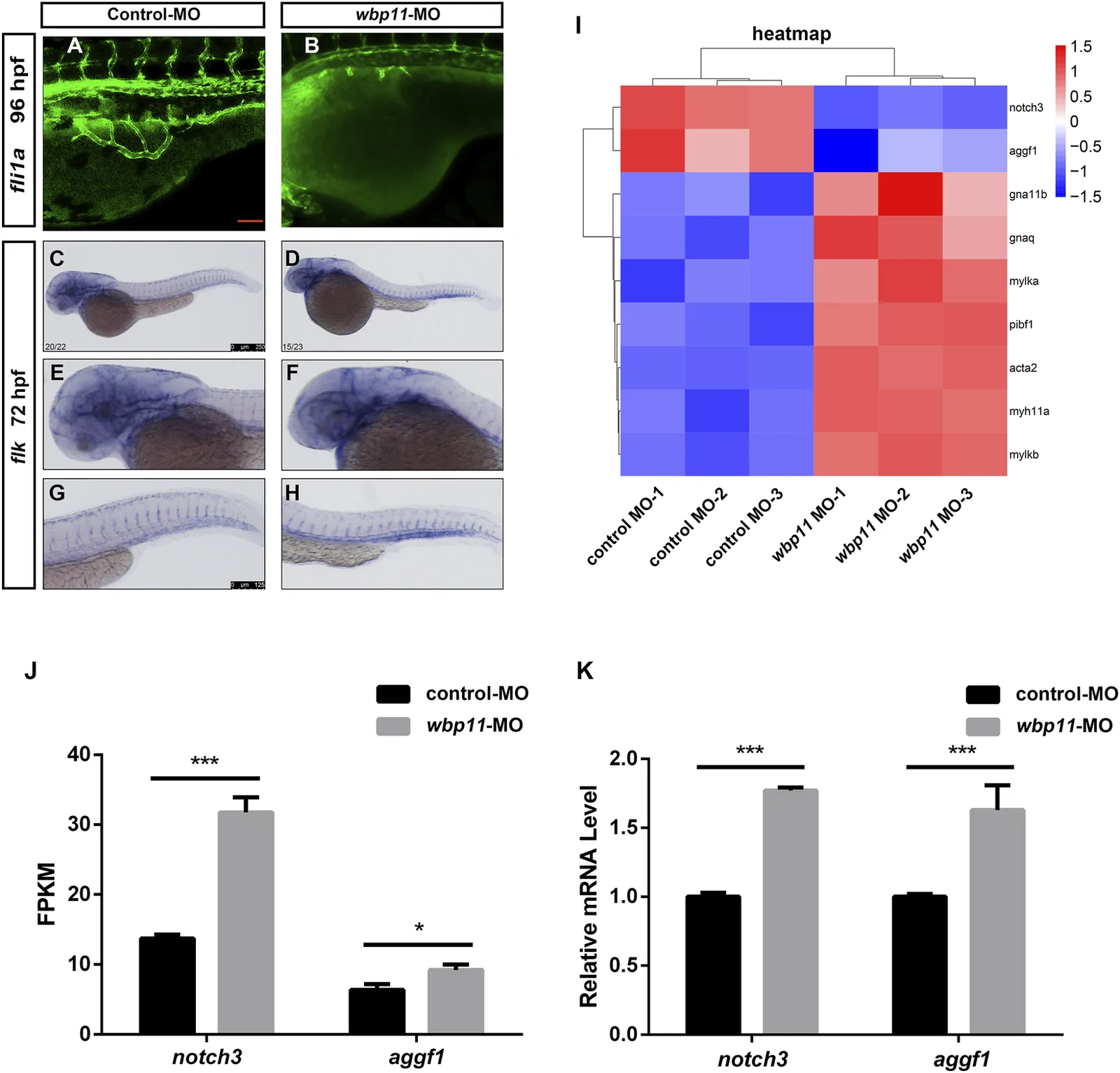
Knockdown of *wbp11* causes vascular defects in zebrafish. **(A,B)** Fluorescence microscopy observation of subintestinal vessels (SIVs) morphology in *Tg(fli1a:EGFP)* zebrafish larvae at 96 hpf. Red scale bar: 100 μm (applies to **(A,B)**). **(C–H)** Whole-mount *in situ* hybridization of zebrafish larvae at 72 hpf using the *flk* probe to visualize the vasculature. **(C,D)** Overview of the entire vascular system. **(E,F)** Cranial vascular network. **(G,H)** Truncal vasculature. Black scale bar: 250 μm (applies to **(C,D)**); black scale bar: 125 μm (applies to **(E–H)**). **(I)** Heatmap analysis of vascular-related differentially expressed genes (DEGs). Blue indicates downregulated genes, and red indicates upregulated genes. **(J)** FPKM values of representative vascular genes *notch3* and *aggf1* (n = 3; biologically independent experiments, each performed with 50 pooled embryos per group. Data are presented as mean ± SEM. *p < 0.05, ***p < 0.001). **(K)** qPCR analysis of transcript levels of representative vascular genes *notch3* and *aggf1* (n = 3; biologically independent experiments, each performed with 50 pooled embryos per group. Data are presented as mean ± SEM. ***p < 0.001).

To evaluate the impact of *wbp11* knockdown on vascular development, we utilized the Tg (*fli1a*:EGFP) transgenic line, which specifically labels vascular endothelial cells. At 96 hpf, larvae in the Control-MO group exhibited intact vascular networks, whereas those in the *wbp11*-MO group displayed varying degrees of angiogenic defects, primarily characterized by hypoplasia of the subintestinal vessels (SIV) and reduced branching (Figures 6A,B; Table 8; Supplementary Figure S4B). Whole-mount *in situ* hybridization for the vascular endothelial marker gene *flk* revealed that larvae in the *wbp11*-MO group had reduced cranial vessel density and a narrowed region of the caudal venous plexus (Figures 6C–H), suggesting that *wbp11* knockdown impairs angiogenesis in zebrafish. Heatmap analysis demonstrated significant alterations in the expression profiles of vascular development-related genes upon *wbp11* knockdown (Figure 6I). Validation of the angiogenesis associated genes *notch3* and *aggf1* using FPKM values and qPCR showed that *wbp11* knockdown led to dysregulation of these vascular related genes compared to the Control-MO group (Figures 6J,K). Furthermore, co-injection of *wbp11* mRNA reversed the transcriptional changes of *notch3* and *aggf1* in the *wbp11*-MO group (Supplementary Figure S5). Collectively, these results indicate that *wbp11* is essential for normal vascular development in zebrafish.

**TABLE 8:** Statistics on SIV malformations rate after MO injection.

Group	Proportion of SIV malformations in abnormal embryos (%)
Control-MO	2.45
	2.55
	3.19
*wbp11*-MO	66.54
	63.48
	54.73

Data were pooled from three independent injection experiments. Total N = 590 embryos for the Control-MO group and N = 632 embryos for the *wbp11*-MO group.

### 3.6 Knockdown of *wbp11* leads to abnormal spine and notochord development in zebrafish

**FIGURE 7:**
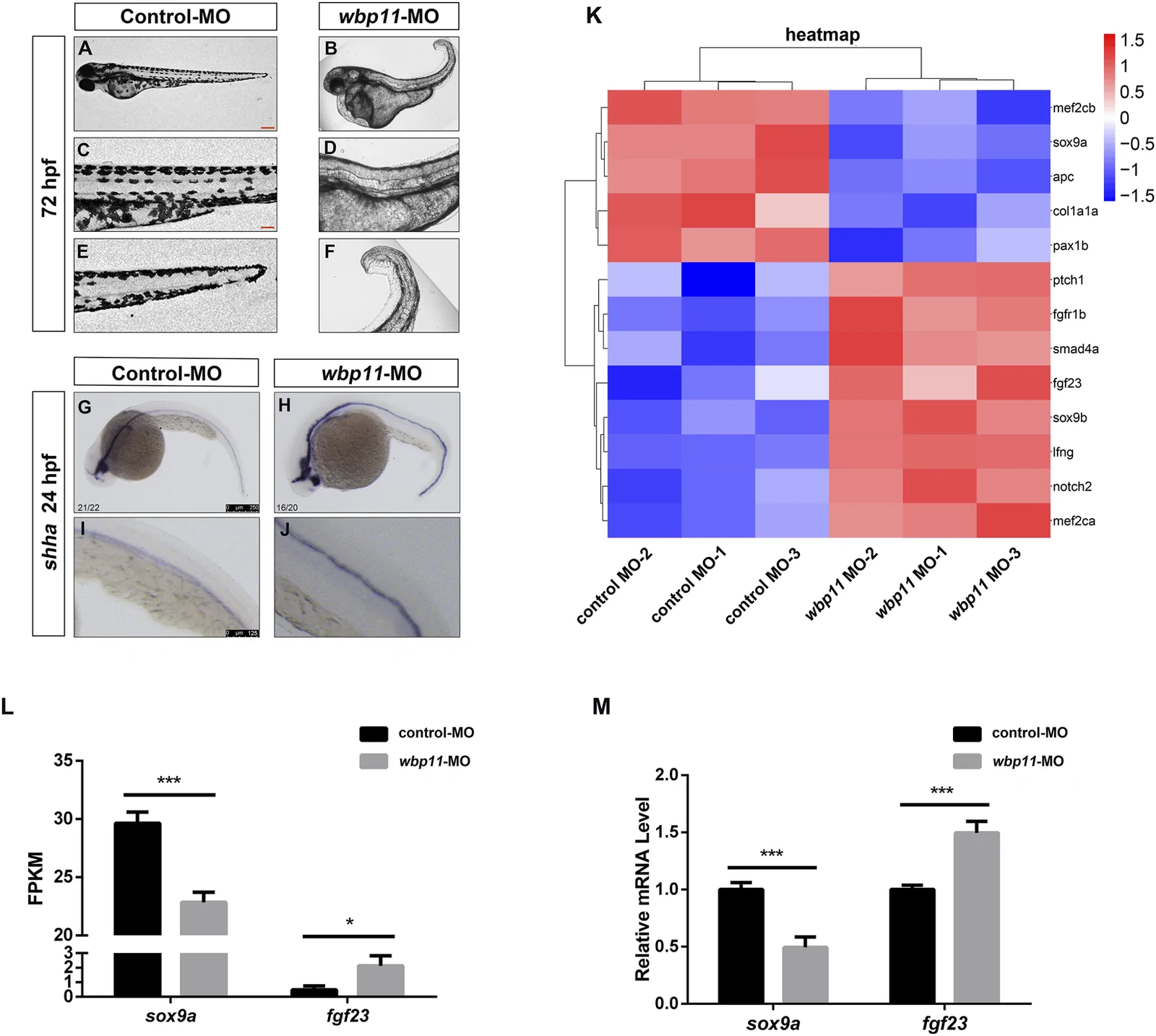
Knockdown of *wbp11* causes spinal defects in zebrafish. **(A–F)** Bright field microscopy observation of spinal morphology in zebrafish larvae. Red scale bar: 100 μm (applies to **(A,B)**); red scale bar: 50 μm (applies to **(C–F)**). **(G–J)** Whole-mount *in situ* hybridization of zebrafish larvae at 24 hpf using the *shha* probe to assess spinal development. Scale bar: 250 μm (applies to **(G,H)**); scale bar: 125 μm (applies to **(I,J)**). **(K)** Heatmap analysis of spine related differentially expressed genes (DEGs). Blue indicates downregulated genes, and red indicates upregulated genes. **(L)** FPKM values of representative spine-related genes *sox9a* and *fgf23* (n = 3; biologically independent experiments, each performed with 50 pooled embryos per group. Data are presented as mean ± SEM. *p < 0.05, ***p < 0.001). **(M)** qPCR analysis of transcript levels of representative spine-related genes *sox9a* and *fgf23* (n = 3; biologically independent experiments, each performed with 50 pooled embryos per group. Data are presented as mean ± SEM. ***p < 0.001).

Spinal curvature was observed in embryos injected with *wbp11*-MO as early as 24 hpf, with the phenotype being most pronounced at 72 hpf; therefore, this time point was selected for analysis. Stereomicroscopic observation revealed that larvae in the Control-MO group exhibited a straight spine with normal development, whereas those in the *wbp11*-MO group displayed spinal curvature and a curled tail (Figures 7A,B; Table 9; Supplementary Figure S4C). Higher magnification further showed that, compared to the Control-MO group, larvae in the *wbp11*-MO group exhibited an irregularly curved notochord structure (Figures 7C,D) and pronounced tail curling (Figures 7E,F). Whole mount *in situ* hybridization for the notochord marker gene shha showed a smooth and continuous notochord in Control-MO embryos, whereas the notochord in *wbp11*-MO embryos exhibited multiple bends (Figures 7G–J). Heatmap analysis of differentially expressed genes (DEGs) from the transcriptome data revealed significant alterations in the expression of spine development-related genes following *wbp11* knockdown (Figure 7K). The chondrogenesis associated gene *sox9a* and the skeletal development-related gene *fgf23* were selected for validation; both FPKM values and qPCR results showed aberrant expression of these genes in the *wbp11*-MO group compared to the Control-MO group (Figures 7L,M). Furthermore, co-injection of *wbp11* mRNA reversed the transcriptional changes of *sox9a* and *fgf23* in the *wbp11*-MO group (Supplementary Figure S5). Collectively, these findings indicate that *wbp11* plays a critical role in spine and notochord morphogenesis.

**TABLE 9:** Statistics on spinal defects rate after MO injection.

Group	Proportion of spinal defects in abnormal embryos (%)
Control-MO	4.37
	5.67
	3.84
*wbp11*-MO	74.62
	70.48
	64.05

Data were pooled from three independent injection experiments. Total N = 590 embryos for the Control-MO group and N = 632 embryos for the *wbp11*-MO group.

### 3.7 Knockdown of *wbp11* causes fin hypoplasia in zebrafish

**FIGURE 8:**
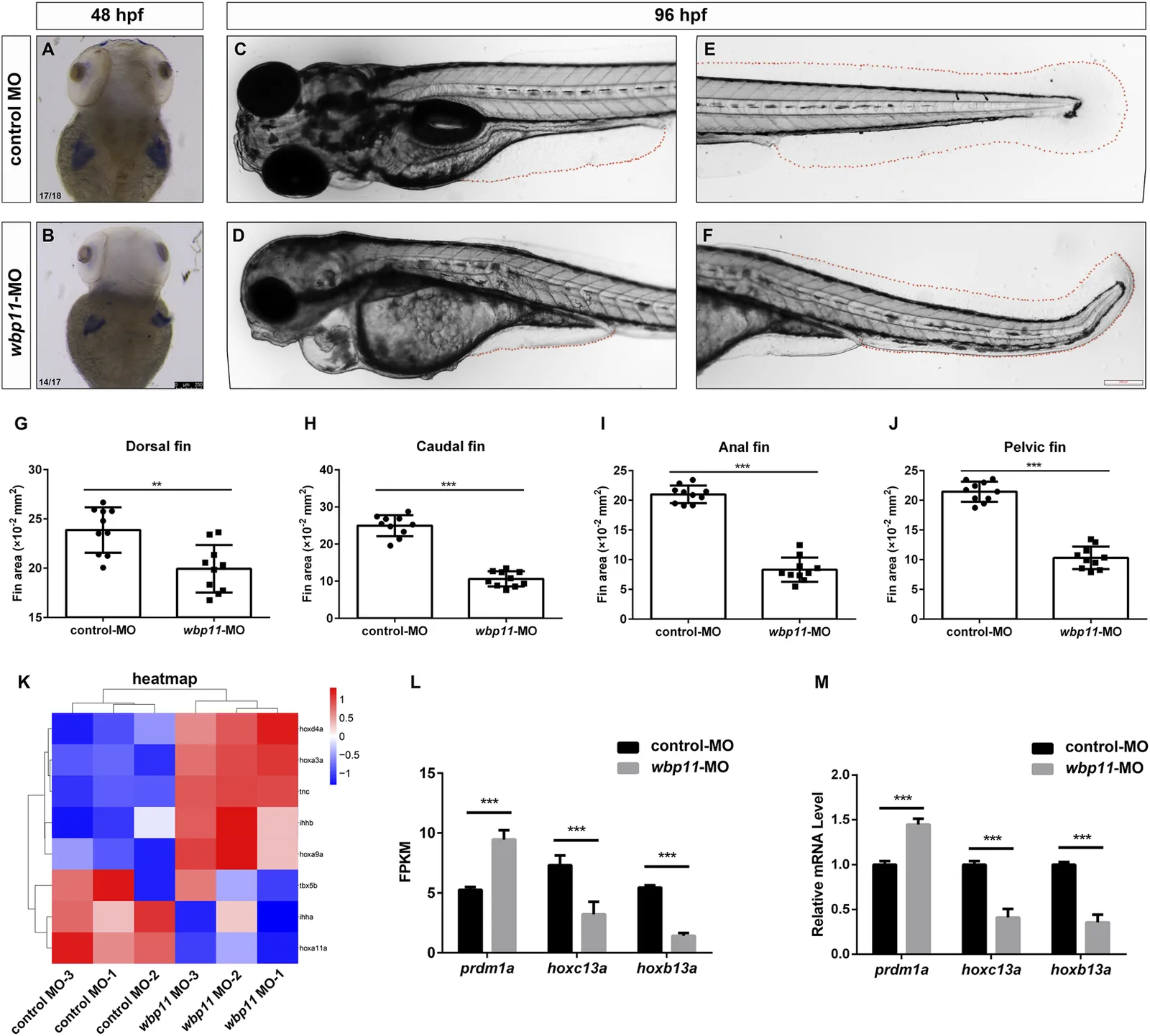
Knockdown of *wbp11* causes fin developmental defects in zebrafish. **(A,B)** Whole-mount *in situ* hybridization of zebrafish larvae at 48 hpf using the *tbx5a* probe to assess fin development. Scale bar: 250 μm (applies to **(A,B)**). **(C–F)** Bright field microscopy observation of fin morphology in zebrafish larvae at 96 hpf. Scale bar: 200 μm (applies to **(C–F)**). **(G)** Quantification of dorsal fin area (n = 10; **: p < 0.01). **(H)** Quantification of caudal fin area (n = 10; ***p < 0.001). **(I)** Quantification of anal fin area (n = 10; ***: p < 0.001). **(J)** Quantification of pelvic fin area (n = 10; ***p < 0.001). **(K)** Heatmap analysis of fin-related differentially expressed genes (DEGs). Blue indicates downregulated genes, and red indicates upregulated genes. **(L)** FPKM values of representative fin-related genes *prdm1a*, *hoxc13a*, and *hoxb13a* (n = 3; biologically independent experiments, each performed with 50 pooled embryos per group. Data are presented as mean ± SEM. ***p < 0.001). **(M)** qPCR analysis of transcript levels of representative fin related genes *prdm1a*, *hoxc13a*, and *hoxb13a* (n = 3; biologically independent experiments, each performed with 50 pooled embryos per group. Data are presented as mean ± SEM. ***p < 0.001).

To investigate the impact of *wbp11* knockdown on fin development, we first examined the expression of the pectoral fin marker gene *tbx5a* by *in situ* hybridization at 48 hpf. The results showed that the *tbx5a* signal in the pectoral fin region was markedly reduced in *wbp11*-MO larvae compared to the Control-MO group (Figures 8A,B). Morphological observations revealed that the pectoral fin area was significantly smaller in *wbp11*-MO larvae than in the control group (Figures 8C–F). Quantitative analysis of fin areas further demonstrated that the pectoral, pelvic, anal, and caudal fins were all significantly reduced in size in the *wbp11*-MO group (Figures 8G–J). Heatmap analysis of differentially expressed genes (DEGs) revealed marked alterations in the expression profiles of fin development-related genes following *wbp11* knockdown (Figure 8K). The fin ray development-associated genes *prdm1a*, *hoxc13a*, and *hoxb13a* were selected for validation; both FPKM values and qPCR results showed that *prdm1a* expression was significantly upregulated, whereas *hoxc13a* and *hoxb13a* were significantly downregulated in the *wbp11*-MO group compared to the Control-MO group (Figures 8L,M). Furthermore, co-injection of *wbp11* mRNA reversed the transcriptional changes of *prdm1a*, *hoxc13a*, and *hoxb13a* in the *wbp11*-MO group (Supplementary Figure S5). Collectively, these findings indicate that *wbp11* is essential for normal fin development in zebrafish.

### 3.8 Conclusion

In summary, this study establishes, for the first time, a zebrafish model of *wbp11* knockdown and systematically reveals the multiple roles of *wbp11* during early embryonic development. Knockdown of *wbp11* leads to developmental delay, a significantly increased malformation rate, and a spectrum of defects including cardiac abnormalities (pericardial edema and impaired heart looping), angiogenic defects (hypoplasia of the subintestinal vessels and reduced cranial vasculature), spinal curvature with notochord structural abnormalities, and fin hypoplasia phenotypes, and transcriptional dysregulation of kidney developmental marker genes (Supplementary Figures S5, S6), which together partially recapitulate the core clinical features of human VACTERL association. Transcriptome analysis further demonstrates that *wbp11* knockdown affects calcium signaling, adrenergic signaling in cardiomyocytes, and the expression of multiple development related genes, thereby providing molecular insights into the underlying pathogenic mechanisms. This study offers a new animal model for investigating the pathogenesis of VACTERL association and lays an important foundation for future exploration of the regulatory networks involving WBP11 in organ development.

## 4 Discussion

VACTERL association is a non-random constellation of congenital anomalies affecting multiple organ systems, and its etiology is complex, with genetic factors playing a significant role (Solomon, 2018). Although *WBP11* has been established as a candidate causative gene for this association, and human mutation carriers exhibit multi-system abnormalities including cardiac, vertebral, and renal defects, existing mouse models only partially recapitulate the vertebral and renal anomalies and fail to recapitulate the cardiac defects (Martin et al., 2020). This limitation has substantially hampered a deeper understanding of the pathogenic mechanisms by which *WBP11* contributes to VACTERL association, highlighting the urgent need for alternative model systems to fill this gap. In the present study, using morpholino-mediated knockdown in zebrafish, we systematically uncovered multiple roles for *wbp11* during early embryonic development and demonstrated that *wbp11* deficiency partially recapitulates the core organ phenotypes of VACTERL association, thereby providing a new platform for mechanistic studies of this disorder.

Zebrafish have emerged as a powerful model organism for studying human developmental disorders, sharing approximately 70% genomic homology with humans, and 84% of known human disease associated genes have orthologs in zebrafish (Howe et al., 2013). This model offers distinct advantages, including embryonic transparency, ex utero development, ease of *in vivo* observation, and amenability to genetic manipulation (Kimmel, 1972; Haffter et al., 1996; Detrich et al., 1999; Kumari et al., 2018). Additionally, zebrafish represent a suitable system for drug screening to identify potential pharmacological interventions (Dooley and Zon, 2000). In this study, bioinformatics analysis confirmed that zebrafish wbp11 shares a high degree of amino acid sequence homology with its human and mouse orthologs, suggesting strong functional conservation throughout evolution (Figure 1). Expression profiling revealed that *wbp11* is a maternal gene, with transcripts present in fertilized eggs, peaking at the gastrula stage and persisting thereafter. Whole-mount *in situ* hybridization further demonstrated relatively higher signal intensity in the head, skeletal muscle, heart, and pectoral fin buds, implying a role in the developmental regulation of multiple systems, including the nervous, musculoskeletal, cardiovascular, and limb systems (Figure 2). Notably, this expression pattern closely aligns with the organ systems affected in human VACTERL association, establishing a critical foundation for functional studies and for the screening and development of relevant disease models. Thus, the zebrafish provides a compelling new animal model for investigating VACTERL association.

In this study, we effectively knocked down *wbp11* expression using morpholino technology and observed a spectrum of developmental defects that closely resemble VACTERL association, including cardiac abnormalities, angiogenic defects, spinal curvature, and fin hypoplasia. Cardiac abnormalities were among the most prominent findings in this study. *wbp11* knockdown embryos exhibited morphological defects such as pericardial edema and impaired heart looping, which are pathologically analogous to the congenital heart defects including ventricular septal defects and tetralogy of Fallot observed in human VACTERL association (Figure 5). Of note, the cardiac phenotype was absent in heterozygous *Wbp11* knockout mice, which may reflect species-specific differences in cardiac developmental regulatory networks and further underscores the unique value of the zebrafish model in this context. Dysregulation of the cardiomyocyte specific genes *actc1a*, *myh7*, and *tpm1* provided additional evidence that *wbp11* is involved in the regulation of cardiomyocyte differentiation and contractile function (Figure 5). Enrichment analysis of the transcriptome data revealed significant perturbation of the adrenergic signaling pathway in cardiomyocytes, suggesting that *wbp11* may influence cardiac morphogenesis by modulating the expression of genes within this pathway. Angiogenic defects represent another important phenotype revealed in this study. *wbp11* knockdown resulted in hypoplasia of the subintestinal vessels, reduced cranial vessel density, and narrowing of the caudal venous plexus, accompanied by downregulated expression of the vascular marker gene *flk* and significant dysregulation of the angiogenesis-related genes *notch3* and *aggf1* (Figure 6). The angiogenic factor AGGF1 and Notch signaling play critical roles in vascular endothelial cell differentiation, vascular development, and angiogenesis (Gridley, 2007; Lv et al., 2025). The downregulation of these genes observed in our study suggests that *wbp11* may contribute to vascular defects by affecting these key angiogenic regulators. Although vascular anomalies, such as single umbilical artery, are recognized as part of the VACTERL spectrum (Ritter et al., 2022), they have received limited attention in previous studies on WBP11. Our findings thus provide novel experimental evidence linking *wbp11* to the vascular phenotypes associated with VACTERL association. Abnormal spine and notochord development in *wbp11* knockdown embryos was manifested as spinal curvature and notochord bending, accompanied by dysregulated expression of the notochord marker gene *shha*. Dysregulation of *sox9a* and *fgf23* further supported a role for *wbp11* in skeletal development (Figure 7). This finding is highly consistent with the vertebral anomalies observed in human VACTERL patients and in mouse models, indicating that the function of wbp11 in axial skeletal development is evolutionarily conserved. Fin hypoplasia was characterized by significant reductions in the areas of the pectoral, pelvic, anal, and caudal fins, downregulation of the fin marker gene *tbx5a*, and aberrant expression of the fin ray development-related genes *prdm1a*, *hoxc13a*, and *hoxb13a* (Figure 8). Hox gene family members play central roles in establishing the patterning along the limb axis, and their dysregulation can lead to limb malformations (Young et al., 2019). Limb anomalies represent one of the core diagnostic criteria for human VACTERL association (Solomon, 2011). The fin defects observed in zebrafish in this study are evolutionarily homologous to human limb abnormalities (Hawkins et al., 2021), further supporting a conserved role for *wbp11* in limb development. Furthermore, bioinformatics analysis of the transcriptomic data combined with qPCR validation revealed that the transcriptional levels of several renal development related genes were altered, with key marker genes including *lhx1a*, *cdh17* and *tfap2a* showing significant downregulation, preliminarily indicating that *wbp11* deficiency impairs renal development (Supplementary Figure S6). Collectively, these findings demonstrate a close molecular association between *wbp11* deficiency and the gene regulatory networks underlying the organ defects characteristic of VACTERL association.

Transcriptome sequencing analysis provided important insights into the molecular mechanisms underlying the multi-organ developmental defects caused by *wbp11* knockdown. KEGG enrichment analysis revealed that the differentially expressed genes were significantly enriched in pathways including neuroactive ligand receptor interaction, calcium signaling, adrenergic signaling in cardiomyocytes, and retinol metabolism (Figure 4). Of note, as a spliceosome associated protein, WBP11 participates in pre-mRNA splicing (Park et al., 2020; Wei et al., 2024). The multi-organ developmental defects and widespread dysregulation of genes involved in multiple signaling pathways observed in this study may, in part, result from global or tissue specific splicing abnormalities induced by *wbp11* knockdown. For instance, splicing variants of key factors within the calcium or adrenergic signaling pathways could alter their expression levels or functional activities, thereby affecting the development of the corresponding organs. We further performed differential alternative splicing analysis on the existing RNA-seq data and identified various alternative splicing events (Supplementary Figure S3), providing direct evidence that the splicing function of Wbp11 is affected upon *wbp11* knockdown. Nevertheless, the specific splicing targets of wbp11 and their functional contributions to individual organ phenotypes remain to be fully elucidated.

The major innovations of this study are as follows. First, we established a zebrafish *wbp11* knockdown model and systematically characterized its functions in the development of multiple organs, including the heart, vasculature, spine, and fins. Second, we demonstrated that this model partially recapitulates the core phenotypes of human VACTERL association, thereby addressing the limitation of existing mouse models, which fail to reproduce the cardiac defects. Third, integrated transcriptome sequencing analysis revealed multiple signaling pathways affected by *wbp11* knockdown, providing important clues for further mechanistic investigations.

Several limitations of this study should be acknowledged. First, morpholino mediated gene knockdown may be associated with off-target effects. To address this concern, we optimized the injection concentration through dose-gradient experiments, validated the knockdown efficiency by Western blotting, and performed rescue experiments by co-injecting *wbp11* mRNA, which successfully reversed the defective phenotypes caused by *wbp11* deficiency (Figure 3; Supplementary Figures S2, S5; Supplementary File S1). Reassuringly, we have previously established a stable *wbp11* knockout zebrafish line using CRISPR/Cas9 technology, and this work has been published as a Chinese language article focusing primarily on the generation of the line and preliminary phenotypic observations. The phenotypic consistency between the CRISPR/Cas9 stable knockout model and the MO-mediated knockdown model in the present study provides compelling evidence supporting the specificity of the MO-induced phenotypes (You et al., 2023). Furthermore, we intercrossed the *wbp11* heterozygous knockout line and analyzed the malformation rates and associated phenotypes in the progeny. Preliminary results indicate that *wbp11* heterozygous mutants can recapitulate the VACTERL association phenotypes induced by MO knockdown (Supplementary File S2). Second, our phenotypic analysis focused primarily on early embryonic developmental stages, and whether *wbp11* deficiency affects long term maintenance of organ function remains unexplored. Third, although transcriptome analysis indicated dysregulation of multiple signaling pathways, the direct splicing targets of wbp11 given its role as a splicing factor remain to be elucidated through in-depth analysis of alternative splicing events by RNA-seq.

In summary, this study successfully established a *wbp11* knockdown zebrafish model and demonstrated that it partially recapitulates the multi-organ developmental defects associated with VACTERL association, including cardiac, vascular, spinal, limb, and kidney abnormalities, thereby providing a new animal platform for elucidating the pathogenesis of this disorder. Transcriptome analysis revealed dysregulation of calcium signaling, adrenergic signaling, and multiple development related genes, offering initial insights into the molecular functions of *wbp11*. Future studies may extend these findings through the following directions: 1) utilizing the existing *wbp11* knockout line to further confirm phenotypic specificity and enable long term follow-up; 2) performing in depth RNA-seq splicing analysis to identify alternative splicing events directly regulated by *wbp11* and their downstream target genes; 3) employing tissue specific knockout approaches to dissect the cell autonomous roles of *wbp11* in the development of distinct organs; and 4) utilizing this model for small molecule compound screening to explore potential therapeutic strategies for VACTERL association. Collectively, this work not only deepens our understanding of the functions of *WBP11* in embryonic development but also provides new perspectives and tools for mechanistic studies and therapeutic exploration in VACTERL association.

## Data Availability

The original contributions presented in this study are publicly available. The RNA-seq data can be found here: NCBI Gene Expression Omnibus (GEO), accession number GSE341689.

## Supplementary material

SUPPLEMENTARY FIGURE S1 Quantification of wbp11 protein expression and malformation rates in zebrafish embryos. **(A)** Densitometric quantification of wbp11 protein expression levels in zebrafish embryos injected with Control-MO or *wbp11*-MO at 72 hpf. Data are presented as mean ± SEM from three independent experiments (n = 3; biologically independent experiments, each performed with 100 pooled embryos per group. **: p < 0.01, ***: p < 0.001). **(B)** Densitometric quantification of wbp11 protein expression levels in zebrafish embryos injected with Control-MO or *wbp11*-MO at 96 hpf. Data are presented as mean ± SEM from three independent experiments (n = 3; biologically independent experiments, each performed with 100 pooled embryos per group. **: p < 0.01, ***: p < 0.001). **(C)** Statistical analysis of the overall malformation rate in zebrafish embryos at 72 hpf following *wbp11*-MO injection. Data were pooled from three independent injection experiments. (n = 3; ***: p < 0.001). **(D)** Statistical analysis of the proportion of different malformation phenotypes in zebrafish embryos at 72 hpf following *wbp11*-MO injection. Data were pooled from three independent injection experiments.

SUPPLEMENTARY FIGURE S2
*wbp11* knockdown induces severe developmental defects, which are partially rescued by wild-type mRNA co-injection. **(A)** Representative lateral view of a Control-MO injected embryo at 72 hpf displaying normal morphology and swim bladder inflation. **(B–D)** Progressive phenotypic severity in *wbp11*-MO injected embryos at 72 hpf: **(B)** severe phenotype characterized by extensive pericardial edema and a linearized heart tube; **(C)** moderate phenotype showing mild cardiac edema and spinal curvature; and **(D)** mild phenotype exhibiting a slightly curved body axis. **(E)** Lateral view of a representative embryo co-injected with *wbp11*-MO and *wbp11* mRNA, showing significant morphological rescue to near-normal appearance.

SUPPLEMENTARY FIGURE S3 Differential alternative splicing analysis of RNA-seq data upon *wbp11* knockdown. **(A)** Analysis of novel transcripts identified from the transcriptomic data upon *wbp11* knockdown. The horizontal axis indicates the various databases, and the vertical axis represents the number of annotated novel transcripts. **(B)** Differential alternative splicing analysis of the transcriptomic data upon *wbp11* knockdown. KnownEventDiff, known transcripts exhibiting differential alternative splicing; KnownEventNoDiff, known transcripts without differential alternative splicing; NovelEventDiff, novel transcripts exhibiting differential alternative splicing; NovelEventNoDiff, novel transcripts without differential alternative splicing. The horizontal axis displays the five types of alternative splicing events identified by rMATS, and the vertical axis indicates the number of transcripts. **(C)** Sashimi plot showing differential splicing of the *tnnt3b* gene between Control-MO and *wbp11*-MO samples. The plot visualizes exon structure (black bars), intron retention, junction read counts (numbers on arcs), and expression levels (RPKM). The IncLevel values reflect the relative abundance of the exon-included isoform, which appears to be decreased in the *wbp11*-MO group compared to the Control-MO.

SUPPLEMENTARY FIGURE S4 Statistical analysis of the proportion of organ malformations in malformed zebrafish embryos. **(A)** Proportion of cardiac malformations in malformed zebrafish embryos at 72 hpf following *wbp11*-MO injection. **(B)** Proportion of subintestinal vessel (SIV) malformations in malformed zebrafish embryos at 72 hpf following *wbp11*-MO injection. **(C)** Proportion of spinal malformations in malformed zebrafish embryos at 72 hpf following *wbp11*-MO injection. Data are presented as mean ± SEM from three independent experiments (n = 3; ***: p < 0.001).

SUPPLEMENTARY FIGURE S5 qPCR analysis of ten target genes in 72 hpf embryos demonstrating rescue by wild-type mRNA co-injection. Compared to the *wbp11*-MO group (black bars), the rescue group (gray bars) showed significantly increased expression of five genes (*myh7*, *tpm1*, *sox9a*, *hoxc13a* and *hoxb13a*) and significantly decreased expression of the remaining five genes (*actc1a*, *aggf1*, *notch3*, *fgf23* and *prdm1a*). (n = 3; biologically independent experiments, each performed with 10 pooled embryos per group). Data represent mean ± SD; **p < 0.01,***p < 0.001 (one-way ANOVA). Scale bar = 100 μm.

SUPPLEMENTARY FIGURE S6 The knockdown of *wbp11* modifies the transcriptional levels of renal related genes in zebrafish. **(A)** Heatmap analysis of renal related differentially expressed genes (DEGs). Blue indicates downregulated genes, and red indicates upregulated genes. **(B)** FPKM values of representative renal-related genes *cdh17*, *tfap2a*, and *lhx1a* (n = 3; biologically independent experiments, each performed with 50 pooled embryos per group. Data are presented as mean ± SEM. #: p > 0.05; **: p < 0.01; ***: p < 0.001). **(C)** qPCR analysis of transcript levels of representative renal-related genes *cdh17*, *tfap2a*, and *lhx1a* (n = 3; biologically independent experiments, each performed with 50 pooled embryos per group. Data are presented as mean ± SEM. *: p < 0.05; **: p < 0.01).
