# Supplementary material for: Loss of wbp11 causes multi-system developmental defects: a zebrafish model of VACTERL association
Source: Front Cell Dev Biol. 2026 Aug 6;14:1846751. doi: 10.3389/fcell.2026.1846751 (PMC13490766; doi:10.3389/fcell.2026.1846751)
Supplement: Supplementary file 1 [file DataSheet1.docx]

Sup-table 1. Embryonic mortality dynamics following *wbp11* morpholino knockdown and mRNA rescue.

| **Group** | **Total injected (n)** | **Survived at 72 hpf (n)** | **Dead (n)** | **Death at 12 hpf** | **Death at 24 hpf** | **Death at 36 hpf** | **Death at 48 hpf** | **Death at 72 hpf** |
| --- | --- | --- | --- | --- | --- | --- | --- | --- |
| **Control-MO** | 156 | 143 | 13 | 6 | 5 | 2 | 0 | 0 |
| ***wbp11*-MO** | 171 | 102 | 69 | 21 | 23 | 17 | 3 | 5 |
| ***wbp11*-MO + mRNA (rescue)** | 136 | 104 | 32 | 13 | 10 | 7 | 2 | 0 |

**Table Legend: Temporal distribution of embryonic mortality in *wbp11* morphant zebrafish with or without mRNA co-injection.Fertilized eggs were microinjected at the one-cell stage with control morpholino (Control-MO), *wbp11*-targeting morpholino (***wbp11***-MO), or** *wbp11***-MO together with synthetic *wbp11* mRNA (rescue group). Dead embryos were counted at indicated time points (12, 24, 36, 48, 72 hpf).**

**Conclusion**

To address the reviewer's concern regarding the specificity of *wbp11* morpholino induced phenotypes, we performed a rescue experiment by co-injecting *wbp11*-MO together with synthetic wild-type *wbp11* mRNA. As summarized in Sup-table 1 and Table 6, co-injection of *wbp11* mRNA resulted in a marked reduction in overall embryonic mortality and a dramatic decrease in the proportion of morphologically abnormal embryos. Notably, the percentage of phenotypically normal embryos increased from 16.7% in the MO group to 76.0% in the rescue group, approaching the level observed in control-MO injected embryos (91.7%). Furthermore, qPCR analysis revealed that the aberrant expression of ten target genes induced by *wbp11* knockdown was effectively reversed upon mRNA co-injection: five genes (myh7, tpm1, sox9a, hoxc13a, hoxb13a) were significantly upregulated, while the remaining five (actc1a, aggf1, notch3, fgf23, prdm1a) were significantly downregulated in the rescue group relative to the MO group. Collectively, these results demonstrate that co-injection of *wbp11* mRNA is sufficient to rescue the majority of morphological defects and molecular alterations caused by *wbp11*-MO knockdown, thereby further validating the conclusion that *wbp11* plays an essential role in early zebrafish development and that the observed phenotypes are specifically attributable to *wbp11* deficiency rather than off-target effects of the morpholino.
