# Supplementary material for: Loss of wbp11 causes multi-system developmental defects: a zebrafish model of VACTERL association
Source: Front Cell Dev Biol. 2026 Aug 6;14:1846751. doi: 10.3389/fcell.2026.1846751 (PMC13490766; doi:10.3389/fcell.2026.1846751)
Supplement: Supplementary file 2 [file DataSheet2.docx]

Sup-table 2. Embryonic mortality and viability analysis of progeny derived from *wbp11*^+/-^ intercrosse.

| **Total Embryos (n)** | **12 hpf (n)** | **24 hpf (n)** | **36 hpf (n)** | **48 hpf (n)** | **72 hpf (n)** | **Total deceased embryos (n)** | **Total Viable Embryos (n)** |
| --- | --- | --- | --- | --- | --- | --- | --- |
| 85 | 9 | 10 | 3 | 6 | 1 | 29 | 56 |

**Table Legend: A total of 85 fertilized eggs were collected and monitored daily. Zebrafish parental crossbreeding (♂:♀=1:1).**

Sup-table 3. Embryonic phenotypic defects in progeny from *wbp11*^+/-^ intercrosse.

| **Genotype** | **Observed (72 hpf) (n)** | **Mild (n)** | **Moderate (n)** | **Severe (n)** | **No obvious phenotype (n)** |
| --- | --- | --- | --- | --- | --- |
| ***wbp11*^+/+^ (WT)** | 21 | 2 | 0 | 0 | 19 |
| ***wbp11*^+/-^(Het)** | 29 | 14 | 5 | 3 | 7 |
| ***wbp11*^-/-^(Homo)** | 6 | 2 | 1 | 2 | 1 |
| ****Total**** | 56* |  |  |  |  |

Table Legend: **Genotyping was performed at 72 hpf. The observed genotype ratio (Wild-type: Heterozygous: Homozygous mutant = 21:29:6) showed a significant deviation from the expected Mendelian ratio of 1:2:1 (χ² test, p < 0.001), indicating embryonic lethality associated with *wbp11* loss-of-function. Malformations were classified as mild (slight curvature or edema), moderate (overt spinal/kinking, pericardial edema), or severe (grossly malformed body axis, non-beating heart).** * Total Viable Embryos.

Sup-table 4. Summary of major morphological defects observed in *wbp11*^+/-^ embryos at 72 hpf .

| **Defect category** | **Affected / Total** | **Percentage (%)** |
| --- | --- | --- |
| **Spinal defects** | 16 / 29 | 55.2 |
| **Cardiac defects** | 21 / 29 | 72.4 |
| **Fin defects** | 19 / 29 | 65.5 |
| **Vascular defects** | Not assessed | - |

**Table Legend: Defects were scored based on gross morphology under stereomicroscope. Spinal defects included lordosis/kyphosis or kinked tail; cardiac defects included pericardial edema and/or linear heart tube; fin defects referred to reduced caudal fin size or edema. Vascular defects were not assessed due to lack of fluorescent reporter signal.**

****Conclusion****

**In the progeny derived from *wbp11*⁺/⁻intercrosses, homozygous mutants (*wbp11*⁻/⁻) exhibited severe embryonic lethality, with only 6 out of an expected ~21 individuals surviving to 72 hpf (observed vs. expected under Mendelian 1:2:1 ratio: 6 vs. 21.25, χ² test, p < 0.001), and all survivors displayed morphological defects (5/6 with moderate-to-severe abnormalities). Heterozygous embryos (*wbp11*⁺/⁻) also showed reduced viability, but the majority survived and presented a high penetrance of multi-system defects, including spinal (55.2%), cardiac (72.4%), and fin (65.5%) anomalies. This intermediate phenotype in heterozygotes aligns with the predicted haploinsufficiency effect and supports the use of this line as a preliminary disease model for human VACTERL association, where heterozygous carriers often exhibit subclinical or mild manifestations.**
